# Assessing the Diversity of Endogenous Viruses Throughout Ant Genomes

**DOI:** 10.3389/fmicb.2019.01139

**Published:** 2019-05-22

**Authors:** Peter J. Flynn, Corrie S. Moreau

**Affiliations:** ^1^Committee on Evolutionary Biology, The University of Chicago, Chicago, IL, United States; ^2^Department of Science and Education, Integrative Research Center, Field Museum of Natural History, Chicago, IL, United States; ^3^Departments of Entomology and Ecology and Evolutionary Biology, Cornell University, Ithaca, NY, United States

**Keywords:** Formicidae, endogenous viral elements, comparative genome biology, microbes, viral diversity

## Abstract

Endogenous viral elements (EVEs) can play a significant role in the evolution of their hosts and have been identified in animals, plants, and fungi. Additionally, EVEs potentially provide an important snapshot of the evolutionary frequency of viral infection. The purpose of this study is to take a comparative host-centered approach to EVE discovery in ant genomes to better understand the relationship of EVEs to their ant hosts. Using a comprehensive bioinformatic pipeline, we screened all nineteen published ant genomes for EVEs. Once the EVEs were identified, we assessed their phylogenetic relationships to other closely related exogenous viruses. A diverse group of EVEs were discovered in all screened ant host genomes and in many cases are similar to previously identified exogenous viruses. EVEs similar to ssRNA viral proteins are the most common viral lineage throughout the ant hosts, which is potentially due to more chronic infection or more effective endogenization of certain ssRNA viruses in ants. In addition, both EVEs similar to viral glycoproteins and retrovirus-derived proteins are also abundant throughout ant genomes, suggesting their tendency to endogenize. Several of these newly discovered EVEs are found to be potentially functional within the genome. The discovery and analysis of EVEs is essential in beginning to understand viral–ant interactions over evolutionary time.

## Introduction

Endogenous viral elements (EVEs), or viral fossils, are whole or fragmented viral sequences integrated into host genomes after viral infection, which can then propagate through the germline. The majority of research conducted on endogenous viruses centers around retroviruses, which has led to discoveries demonstrating that these viruses could play a role in the evolution of their hosts. EVEs were found to be potentially important in the evolution of placental mammals as well as in the resistance to a variety of diseases (Feschotte and Gilbert, 2012; Grasis, 2017).

Endogenous viral elements are created when a duplicate of a double-stranded DNA viral genome is incorporated into the host germline. As part of their replication, retroviruses must manufacture dsDNA intermediates in order to integrate into the host genome. However, DNA and RNA viral endogenization is much less well understood. It is thought to result from inadvertent chromosomal integration such as non-homologous recombination or retrotransposition events (Aiewsakun and Katzourakis, 2015; Figure 1 from Katzourakis and Gifford, 2010). DNA repair machinery from the host cell has the ability to detect viral sequences within the genome (Weitzman et al., 2004). Therefore, EVEs will often be excised from the host genome, though a small number will evade detection. EVEs reach genomic fixation either from neutral evolution or from exaptation, a process whereby EVEs convey beneficial functions distinct from their original purpose to their host (Katzourakis and Gifford, 2010). EVEs will then accrue mutations at the host neutral rate of evolution since they are fixed in the host genome (Katzourakis, 2013). Non-functional EVEs are expected to accumulate mutations at a far slower rate than their exogenous viral counterparts (Aiewsakun and Katzourakis, 2015). EVEs that have been functionally co-opted by the host cell would be expected to have an even slower mutation rate due to being conserved through positive selection. Demographic patterns such as host or viral population size could also affect the viral endogenization. Host species with small effective population sizes (i.e., many mammal species) may contain more neutral EVEs due to the amplified importance of genetic drift (Holmes, 2011).

In recent years, several studies have illustrated how exaptation of EVEs into a host’s genome function in antiviral defense through production of functional proteins (Frank and Feschotte, 2017). For example, in the thirteen-lined ground squirrel (*Ictidomys tridecemlineatus*), an endogenous bornavirus effectively stops infection of exogenous (i.e., viruses which exhibit horizontal transmission from organism to organism) bornaviruses (Fujino et al., 2014). EVEs can have an influential and enduring influence on their host’s evolution and subsistence in a given ecosystem. Therefore, analyzing EVEs across host genomes could help to unravel the complicated evolutionary relationships between viruses and their hosts.

Preliminary evidence suggests that ant genomes contain several clades of EVEs. François et al. (2016) found several putative ant EVE hits when surveying the Parvoviridae viral clade. Li et al. (2015) found a few ant glycoprotein EVEs in a study aimed at discovering arthropod RNA viruses. Dennis et al. (2018) found a single *Pseudomyrmex gracilis* cyclovirus ant EVE in a large survey of Circoviridae EVEs. However, these three studies used a virus-centered approach and only incidentally discovered ant EVEs. Their approach targets a small group of viruses among a wide array of host genomes in order to understand more about that clade of viruses. Conversely, a host-centered approach, in which one surveys a specific group of host genomes for all known viruses, permits the discovery of novel EVEs within those hosts.

Ant species exhibit extremely variable diets (herbivore, predator, and generalist), nesting habitat (arboreal vs. ground), colony structure, and complex and species-specific social behavior (Hölldobler and Wilson, 1990; Lach et al., 2010). Examination of ant EVEs may provide insight into this variability across their evolutionary history. Though all ants share the same RNAi immune response pathway, differences in EVEs across species may signify differential viral pathogen infection rates (Mongelli and Saleh, 2016). Therefore, analysis of EVEs could provide understanding of insect immunity evolution by acting as a reservoir of immune memory. In addition, a survey of EVEs throughout ant genomes could help elucidate the factors shaping the composition of viral communities presently infecting ants. EVEs scattered throughout the genomes of ants could represent a deep branch of the antiviral defense system (Whitfield et al., 2017).

Though there are currently nineteen published ant genomes, there have been no genome-wide studies examining their endogenous viruses. Therefore, the goal of this study is to survey and characterize EVE sequences throughout these nineteen ant genomes. Specifically we aim to address three questions: (1) Do ants exhibit abundant and diverse EVEs throughout their genomes? (2) How are the EVEs found in ant genomes related to exogenous viral clades? (3) Do any of these discovered EVEs exhibit potential for functionality?

## Materials and Methods

A comprehensive bioinformatic pipeline using BLAST was created to screen for EVEs throughout every published ant genome in the NCBI database^1^. There are currently nineteen published ant genomes (Table 1). These assembled genomes are of various sizes ranging from 212.83 megabases to 396.25 megabases. Before each ant genome was screened, scaffolds under 10,000 base pairs (bp) were pruned from the genome with the program CutAdapt (Martin, 2011) to ensure EVE hits were located on the actual genome and not scaffolds potentially created through assembler error or contamination.

**Table 1:** Summary table of ant genomes which includes information on the species, subfamily, accession number from www.NCBI.nlm.nih.gov, total genome length in megabases, nesting habitat (arboreal/ground), diet (fungus, generalist, predatory, and herbivore), and number of EVE hits recovered.

Ant species	Ant subfamily	Accession number	Total	Nesting habitat	Diet	No. putative
			length			EVEs
			(Mb)			Recovered
*Acromyrmex echinatior*	*Myrmicinae*	GCF_000204515.1	295.945	Ground/arboreal	Fungus	23
*Atta cephalotes*	*Myrmicinae*	GCF_000143395.1	317.672	Ground	Fungus	11
*Atta colombica*	*Myrmicinae*	GCF_001594045.1	291.258	Ground	Fungus	10
*Camponotus floridanus*	*Formicinae*	GCF_000147175.1	232.685	Arboreal	Generalist	11
*Cyphomyrmex costatus*	*Myrmicinae*	GCF_001594065.1	300.317	Ground	Fungus	68
*Dinoponera quadriceps*	*Ponerinae*	GCF_001313825.1	259.666	Ground	Predatory	13
*Harpegnathos saltator*	*Ponerinae*	GCF_000147195.1	294.466	Ground	Predatory	17
*Lasius niger*	*Formicinae*	GCA_001045655.1	236.236	Ground	Generalist	2
*Linepithema humile*	*Dolichodirinae*	GCF_000217595.1	219.501	Ground/arboreal	Generalist	19
*Monomorium pharaonis*	*Myrmicinae*	GCF_000980195.1	257.977	Ground	Generalist	13
*Ooceraea biroi*	*Dorylinae*	GCF_000611835.1	212.826	Ground	Predatory	11
*Pogonomyrmex barbatus*	*Myrmicinae*	GCF_000187915.1	235.646	Ground	Herbivore	42
*Pseudomyrmex gracilis*	*Pseudomyrmicinae*	GCF_002006095.1	282.776	Arboreal	Generalist	52
*Solenopsis invicta*	*Myrmicinae*	GCF_000188075.2	396.025	Ground	Generalist	18
*Trachymyrmex cornetzi*	*Myrmicinae*	GCF_001594075.1	369.438	Ground	Fungus	39
*Trachymyrmex septentrionalis*	*Myrmicinae*	GCF_001594115.1	291.747	Ground	Fungus	26
*Trachymyrmex zeteki*	*Myrmicinae*	GCF_001594055.1	267.973	Ground	Fungus	15
*Vollenhovia emeryi*	*Myrmicinae*	GCF_000949405.1	287.901	Ground	Predatory	31
*Wasmannia auropunctata*	*Myrmicinae*	GCF_000956235.1	324.12	Ground/arboreal	Generalist	13


The bioinformatic screen took a conservative approach, which consisted of first executing tblastn on RefSeq viral proteins (and all proteins from Shi et al., 2016) as the query against the specific ant genome as the database. The *e*-value for this tblastn was set at 1e–20 (Blast., 2013). Viral proteins from Shi et al. (2016) were included in the query because this study vastly increased the known viral diversity in insects and was not included in the RefSeq database at the time of this analysis. At the time of the screen, there were 1,149,421 viral proteins included and nineteen ant genomes analyzed. The nucleotide hits from this tblastn were merged with neighboring hits within 10 base pairs into a single sequence. These merged hits were then used as the query for a blastx run against the non-redundant protein database. The *e*-value for this blastx run was set at 0.001. The purpose of this blastx run was to assess if the original hit from the tblastn run was of viral origin. If the best hit was not most similar to a virus, then it was discarded. The final list of putative amino acid EVE hits were then manually pruned to ensure the best hit was also not most similar to a hypothetical protein, but to a viral structural or non-structural protein. Several EVEs were manually concatenated if they were close to each other on the same scaffold and when aligned did not overlap, but instead came from one larger protein fragment.

Once these putative EVEs were identified, the phylogenetic relationships of the EVE hits were inferred from an amino acid alignment of EVE protein hits and their closely related exogenous virus protein sequences, which were determined by most similar BLAST match. EVEs were grouped together based on both the viral protein class (glycoprotein, RNA-dependent RNA polymerase, nucleoprotein, etc.) and viral clade to which it was most similar on the blastx run. These EVEs and their closely related exogenous viral protein sequences were aligned with MAFFT v7.309 (Katoh et al., 2002). For each alignment, the MAFFT program used the algorithm E-INS-I, scoring matrix of BLOSUM62, and a gap open penalty of 1.53. Subsequently, maximum likelihood phylogenies were inferred using the amino acid alignments with RAxMLv8.1.16 (Stamatakis, 2014). The best fit protein substitution model for each alignment was determined using SMS (Lefort et al., 2017). Support for the maximum likelihood (ML) phylogenies was evaluated with 1000 bootstrap replicates.

We assessed if ant genome quality was correlated with the number of EVEs present in the genome. For these analyses, we assessed both the genome directly downloaded from NCBI (pre-clipped) as well as the clipped genome used in the pipeline. By using the Pearson’s product-moment correlation, we compared number of EVEs present in each genome with genome length, number of scaffolds, scaffold N50, number of contigs, and contig N50. BBMap was used to compile the statistical metrics for the clipped genomes (Bushnell, 2014). To understand if size filtering impacted the EVEs we found, we performed a synteny analysis to assess the size and number of annotated host genes on the individual scaffolds in which EVEs were discovered. This analysis was manually performed by examining each scaffold in the NCBI Genome Data Viewer.

To further examine EVE-ant evolutionary relationships, we used BaTS Bayesian tip-association significance testing (Parker et al., 2008; Shi et al., 2018) to assess if the EVE hits from the Mono-Chu glycoprotein phylogeny tend to clump more strongly with a particular ant species than expected solely by chance. The Mono-Chu glycoprotein phylogeny was simplified to include only the 227 ant EVE hits since we were only testing ant host-EVE associations. This test considered host phylogenetic structure at the level of ant species and subfamily. BaTS then estimated an association index (AI) to identify the strength of the association between the Mono-Chu glycoprotein EVE phylogeny and ant host species/subfamily. This AI value was then compared to a null distribution generated using 1000 tree-trip randomizations to infer an Association Index Ratio (observed association index/null association index). A ratio closer to 0 suggests stronger host structure and closer to 1 suggests a weaker host structure. A *p*-Value for the AI was output from the BaTS test derived from the 1000 tree-tip randomizations.

To evaluate the degree of EVE-ant host co-divergence in each ant species, we implemented an event-based co-phylogenetic reconstruction using JANE version 4 (Conow et al., 2010). The simplified EVE hit Mono-Chu glycoprotein phylogeny used for the BaTS test was used for this analysis. In addition, for the host phylogeny we used the ant phylogeny from Nelsen et al. (2018), with the drop.tip function in *ape* to infer a phylogeny with solely the 19 ant species examined in this study. The cost event scheme, or non-co-divergence, for the JANE reconstruction was: co-divergence = 0, duplication = 1, host switch = 1, loss = 1, failure to diverge = 1. The “failure to diverge” parameter refers to occurrences when host speciation is not followed by virus speciation, and the virus remains on both newly speciated hosts. The population size and the number of generations were fixed to 100. The co-divergence significance was determined by contrasting the estimated costs to null distributions calculated from 100 randomizations of host tip mapping. To better visualize these co-divergence patterns, we visualized these associations between the EVEs in the simplified glycoprotein Mono-Chu phylogeny and the EVEs in the simplified Nelsen et al. (2018) ant phylogeny using the cophylo function in phytools to create a tanglegram (Revell, 2012).

The potential functionality of these endogenous viral fragments was assessed through the analysis of the stop codons and nonsense mutation within the EVE hit protein fragments to determine if they possess intact open reading frames (ORFs). Intact ORFs were then inferred to be functional if through manual comparison, the putative EVE protein sequence was within 75 amino acids in length of its most closely related exogenous viral protein.

## Results

**FIGURE 1:**
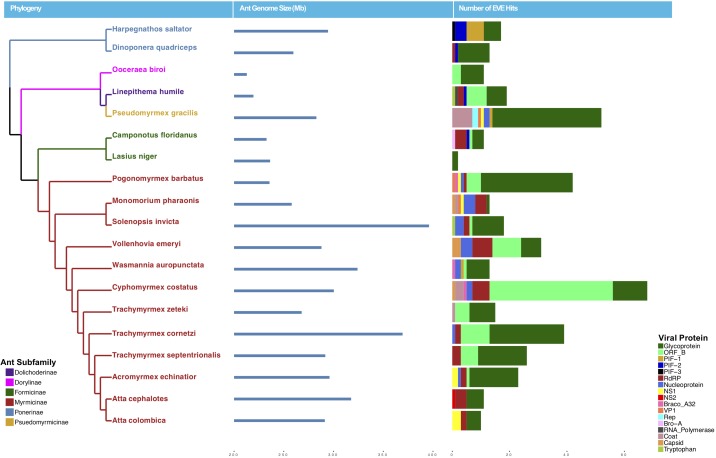
Phylogeny of ants with sequenced genomes used in this study colored by subfamily (left), ant genome size in mb (middle), and number of EVE hits per genome (right). Ant EVE hit numbers are colored by viral protein to which they were found to be most similar in the blastx analysis.

Using a host-centered approach, we screened all 19 available and published ant genomes (on 1/1/2018) with a bioinformatics pipeline detailed in the Material and Methods section above. Once EVE hits were obtained, we assessed their genome-specific differences in abundance and variation. We recovered a total of 434 EVE hits across these 19 ant genomes (Supplementary Tables S1, S2). There is clear variation in the number of EVEs recovered depending on ant species (Figure 1 and Table 1). Table 2 displays the results of the Pearson’s product-moment correlation, comparing the factors representing genome quality with EVE number per genome. Based on the Pearson’s product-moment correlation, none of these factors were significantly correlated with number of EVEs discovered. Supplementary Table S3 contains all genome information used for both the clipped and pre-clipped genomes. For the synteny analysis, we found that 78.57% of the scaffolds containing EVEs were longer than 30,000 base pairs. In addition, 70.74% of these scaffolds had at least one gene annotated from the host. The scaffold length and number of annotated host genes are found in Supplementary Table S1. There were no EVEs that represented an entire viral genome on a single scaffold – instead each EVE hit constituted a single protein or protein fragment.

**Table 2:** Genome quality assessment of factors from the clipped and pre-clipped genomes as correlated with the number of EVEs per genome.

Genome type	Factor	*r*	*p*-Value
Pre-clipped	Genome length	+0.211	0.386
	No. contigs	-0.105	0.669
	Contig N50	+0.415	0.078
	No. scaffolds	-0.160	0.515
	Scaffold N50	-0.177	0.467
Clipped	Genome length	+0.358	0.132
	No. contigs	+0.125	0.611
	Contig N50	-0.101	0.680
	No. scaffolds	-0.124	0.613
	Scaffold N50	+0.091	0.712


*The different genome factors tested included total genome length (Mb), number of contigs, contig N50, number of scaffolds, and scaffold N50. The *r* and *p*-Values derive from the Pearson’s product moment correlation.*

### Viral Phylogenies

A summary of information on the phylogenies presented in the results is shown in Table 3. All the phylogenies described in the subsequent results can be found in Supplementary Figures S1–S23.

**Table 3:** Summary table of viral phylogeny information.

Virus	Virus clade	Protein	No. of	No. of	Supplementary Figure
genome			EVEs	exogenous	
structure				proteins used	
ssRNA	Bunya-Arena	Nucleoprotein	18	18	Supplementary Figure S1
		RdRP	3	12	Supplementary Figure S2
	Hepe-Virga	RdRP	3	15	Supplementary Figure S3
	Mono-Chu	Glycoprotein	227	17	Supplementary Figure S4
		Nucleoprotein	2	12	Supplementary Figure S5
		RdRP	24	30	Supplementary Figure S6
	Narna-Levi	RdRP	2	15	Supplementary Figure S7
	Partiti-Picobirna	Capsid	5	7	Supplementary Figure S8
		RdRP	4	16	Supplementary Figure S9
	Qinvirus	RdRP	3	10	Supplementary Figure S10
	Toti-Chryso	Coat	12	12	Supplementary Figure S11
		RdRP	1	15	Supplementary Figure S12
ssDNA	Circoviridae	rep-associated protein	2	10	Supplementary Figure S13
	Parvoviridae	VP1	3	10	Supplementary Figure S14
		Non-structural protein 1	8	18	Supplementary Figure S15
		Non-structural protein 2	1	12	Supplementary Figure S16
dsDNA	Baculoviridae	Bro-a	1	12	Supplementary Figure S17
		PIF-1	8	18	Supplementary Figure S18
		PIF-2	7	14	Supplementary Figure S19
		PIF-3	1	12	Supplementary Figure S20
	Poxviridae	Tryptophan	2	6	Supplementary Figure S21
		RNA-polymerase RP0147	1	7	Supplementary Figure S22
	Polydnaviridae	Pox A32	3	6	Supplementary Figure S23
ssRNA(RT)	Metaviridae	ORF B	93	NA	NA


*This table includes the virus genome structure, the virus clade and protein to which the EVE hit was most similar, the number of EVEs found per protein phylogeny, the number of exogenous viral proteins used in the phylogenetic reconstruction, and the Supplementary Figure Information. There is no phylogeny for the EVE hits most similar to ssRNA(RT).*

#### ssRNA Viruses

For the names of the RNA viral clades we will be using the nomenclature defined by Shi et al. (2016). Due to the increase in diversity of RNA viruses discovered by Shi et al. (2016), a merger of different viral families and orders into larger super-clades was proposed. For example, the Bunya-Arena clade is comprised of all the viruses from Bunyaviridae, Tenuivirus, Arenaviridae, and Emaravirus. For more detailed information on which smaller viral groups comprise which super-clades, refer to Figure 2 of Shi et al. (2016).

Five different types of proteins found in ssRNA viruses were most similar to the EVE hits: glycoproteins, RNA-dependent RNA polymerases, nucleoproteins, capsid proteins, and coat proteins. All of these proteins have been previously discovered in insect virus genomes (Shi et al., 2016). Glycoproteins were the most commonly found in ant genomes, though only in the Mono-Chu clade. RNA-dependent RNA polymerases (RdRP) were discovered in every clade of ssRNA virus with EVE hits. A few EVE hits similar to nucleoproteins, capsid proteins, and coat proteins were also discovered within distinct ssRNA viral clades. Viral clade phylogeny results are presented in alphabetical order.

##### Bunya-Arena

A total of 17 ant EVEs are most closely related to the exogenous Bunya-Arena viral clade: 16 are most similar to Bunya-Arena nucleoprotein protein fragments and one is most similar to a Bunya-Arena RdRP protein fragment. In the nucleoprotein Bunya-Arena phylogeny, the 16 EVEs are distributed across five clades throughout the phylogeny, though several of these clades are not well-supported and therefore not conclusive (Supplementary Figure S1). Two *P. gracilis* EVEs are in a well-supported clade that is sister to a clade of bunyaviruses (bootstrap value = 100). Three *Vollenhovia emeryi* EVEs hits all cluster together in a weakly supported clade sister to the remaining EVE clades (bootstrap value = 38). Five ant EVEs cluster around Wuhan insect virus 16 in a weakly supported clade (bootstrap value = 16) and five other ant EVEs form a well-supported clade of their own (bootstrap value = 89). Also within this clade, *Monomorium pharaonis* EVE10 is weakly recovered as sister to a Topsovirus clade, which primarily consists of plant viruses (bootstrap value = 29). In the RdRP Bunya-Arena phylogeny, the EVE hit, *Cyphomyrmex costatus* EVE20 is sister to Wuhan insect virus 16 in a well-supported clade (bootstrap = 100; Supplementary Figure S2).

##### Hepe-Virga

In this RdRP Hepe-Virga phylogeny, the three Hepe-Virga-like ant EVEs are all from Myrmicinae genomes and form a well-supported clade with Hubei virga-like virus 1 and 2 (bootstrap value = 100; Supplementary Figure S3).

##### Mono-Chu

**FIGURE 2:**
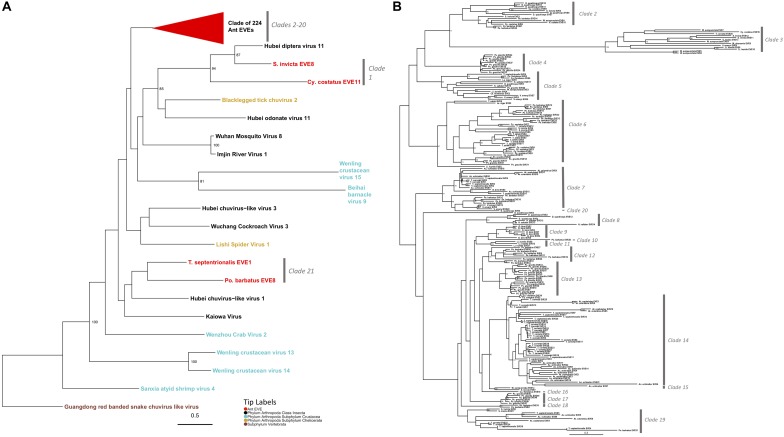
**(A)** Glycoprotein Mono-Chu Phylogeny. The phylogeny was obtained from maximum likelihood analysis of the glycoprotein multiple amino acid alignment, including both ant EVE sequences and closely related exogenous viruses. The best fit protein substitution model was JTT + G + F. ML non-parametric bootstrap values >70 (1000 replicates) are indicated at each node. Scale bar indicates evolutionary distance in substitutions per site. The 224 ant EVEs which comprise their own clade have been collapsed and are represented by the red triangle. The tips are colored by associated host. The gray bars on the right are based on distinct clades of EVE hits. **(B)** Expanded phylogeny representing the clade of 224 ant EVEs which was collapsed in panel **(A)**. ML non-parametric bootstrap values >70 (1000 replicates) are indicated at each node. Scale bar indicates evolutionary distance in substitutions per site. The gray bars on the right are based on distinct clades of EVE hits.

Mono-Chu viruses are the most common viral clade of EVEs within all ant genomes (total of 253 EVEs), primarily due to glycoprotein representing 89.7% of Mono-Chu EVE hits. The other EVE hits are most similar to Mono-Chu nucleoproteins and RdRP. These EVEs came from all 19 ant genomes observed in this study. The Mono-Chu Glycoprotein phylogeny was described in a comprehensive manner within these results due to its use in subsequent analyses. In this phylogeny, there are a total of 21 clades that include ant EVEs (Figure 2A and Supplementary Figure S4). To examine this phylogeny in greater detail refer to http://itol.embl.de/shared/pflynn.

Two clades within this Mono-Chu glycoprotein phylogeny cluster with exogenous viral lineages. Clade 1 is moderately supported and consists of Hubei chuvirus-like virus 1 clustering with two EVE hits (bootstrap value = 55). The other clade is Clade 21, which consists of Hubei diptera virus 11 and two EVE hits (bootstrap value = 94).

The rest of the 224 ant EVEs all fall within ant EVE-specific clades that are not closely related to any exogenous viruses shown in Figure 2B (bootstrap value = 64). Within this part of the phylogeny there are 19 distinct clades, with all but four (Clade 10, 16, 19, and 20) having bootstrap support of 60 or higher. Clade 2 consists of 14 ant EVEs and is moderately well-supported (bootstrap value = 63). Clade 3 is distinct with an extremely well-supported long branch and consists of 13 EVEs (bootstrap value = 100). Clade 4 is highly supported and consists entirely of ten closely related *P. gracilis* EVEs (bootstrap value = 100). Clade 5 is a distinct well-supported clade and consists of 14 EVEs (bootstrap value = 98). Clade 6 is highly supported and 35 EVEs form this clade (bootstrap value = 97). Ten EVEs in this clade cluster together and are all *Pogonomyrmex barbatus* EVEs. Seven EVEs from the *Trachymyrmex* species group together, five *Cyphomyrmex costatus* EVEs group together, five EVEs from *P. gracilis* cluster, and eight EVEs exhibit no host-specific pattern.

Clade 7 is a moderately well supported clade and consists of 23 ant EVEs (bootstrap value = 77). The distinct Clade 8 consists of six EVEs exclusively from the Ponerinae subfamily (bootstrap value = 93). Clade 9 consists only of *Ooceraea biroi* EVEs (bootstrap value = 78). *P. barbatus* EVE25 is a single EVE that forms a not well-supported Clade 10 (bootstrap value = 21). Clade 11 consists of three EVEs from *Linepithema humile* (bootstrap value = 78). The moderately well-supported Clade 12 comprises nine *P. barbatus* EVEs (bootstrap value = 72). Clade 13 is a well-supported clade which consists of 16 *P. gracilis* EVEs (bootstrap value = 91). Clade 14 is a well-supported clade consisted of 48 EVEs from fungus-growing ant genomes (bootstrap value = 81). Clade 15 is a distinct clade which consists of two EVEs from the subfamily Myrmicinae (bootstrap value = 90). Clade 16 consists of a single EVE: *Camponotus floridanus* EVE10 which is sister to the fungus-growing ant Clade 14 (bootstrap value = 37). All six EVEs which form Clade 17 are from the *P. gracilis* genome (bootstrap value = 95). Clade 18 consists of two EVEs from *P. barbatus* (bootstrap value = 97). Clade 19 is a not well-supported clade of 12 EVEs in the Myrmicinae subfamily (bootstrap value = 18). Clade 20 consists of *D. quadriceps* EVE4, (bootstrap value = 37) which is sister to Clades 8–17.

In the Mono-Chu nucleoprotein phylogeny, two EVEs cluster together with Hubei chuvirus-like virus 1 in a well-supported clade (bootstrap value = 99; Supplementary Figure S5).

Eighteen ant EVEs fall into the Mono-Chu RdRP phylogeny (Supplementary Figure S6). Five of these EVEs are nested within a well-supported clade with Orinoco virus (bootstrap value = 93). Two EVEs belong to a well-supported clade with Berant Virus (bootstrap value = 100). *V. emeryi* EVE12 clusters with Beihai rhabdo-like virus 1, a virus infecting Crustaceans in a well-supported clade (bootstrap value = 100). *M. pharaonis* EVE4 belongs in a clade with two vertebrate bornaviruses. *V. emeryi* EVE1 is nested within a clade with Marburgvirus (bootstrap value = 10). Eight EVEs all derived from fungus-growing ant genomes are clustered in a well-supported clade with Shuangao Fly Virus 2 (bootstrap value = 96).

##### Narna-Levi

The two Narna-Levi-like ant EVEs are found in the *L. humile* genome. In the RdRP Narna-Levi phylogeny, these two *L. humile* EVEs are nested within a well-supported clade of narna-like viruses from insect hosts (bootstrap value = 96; Supplementary Figure S7).

##### Partiti-Picobirna

A total of nine ant EVEs are most similar to Partiti-Picobirna viruses related to the RdRP and Capsid proteins. *M. pharaonis* and *V. emeryi* genomes produced hits which are found within both of these protein phylogenies (Supplementary Figures S8, S9).

In the Partiti-Picobirna capsid phylogeny, the five ant EVEs belong to three distinct lineages (Supplementary Figure S8). The first is a well-supported clade which includes three *V. emeryi* EVEs clustering together (bootstrap value = 100) and are sister to Beihai barnacle virus 12 (bootstrap value = 99). The second lineage consists of *M. pharaonis* EVE9 as sister to Wuhan cricket virus 2 (bootstrap value = 85). The third lineage places *C. costatus* EVE12 as sister to Hubei tetragnatha maxillosa virus 8, a virus from an arachnid host, though this position is not as well-supported (bootstrap value = 57).

In the Partiti-Picobirna RdRP phylogeny, the four ant EVEs again fall into three distinct lineages (Supplementary Figure S9). Two EVEs cluster (bootstrap value = 100) and form a clade with Hubei partiti-like virus 29. *D. quadriceps* EVE9 falls out into well-supported clade of Partiti-Picobirna viruses from hosts of *Vespa velutina* (Asian hornet), Coleoptera (beetles), and Lophotrochozoa (snails) (bootstrap = 99). *Solenopsis invicta* EVE9 belongs to a lineage of Partiti-Picobirna viruses with hosts of both insect and chelicerate origin.

##### Qinvirus

The Qinvirus clade was first described in Shi et al. (2016) since the RdRP domains of the discovered viruses were so divergent from any previously known viral clade. *C. floridanus* EVE7 was discovered as an RdRP protein fragment most similar to the Qinvirus clade. In the reconstructed Qinvirus phylogeny, this EVE is sister to Wuhan insect virus 15 (bootstrap value = 78), nested within this larger clade (Supplementary Figure S10).

##### Toti-Chryso

Twelve of the thirteen Toti-Chryso-like ant EVEs are most similar to coat proteins. Seven of these hits are found in the *P. gracilis* genome. From the coat protein Toti-Chryso phylogeny, four of these *P. gracilis* EVEs cluster together as a distinct clade and as a sister clade to Shuangao toti-like virus and Australian anopheles totivirus (bootstrap value = 84; Supplementary Figure S11). Four EVEs belong in a weakly-supported Shaungao toti-like virus/Australian anopheles totivirus clade (bootstrap value = 42). Two EVEs cluster within a well-supported clade with *Leptopilina boulardi* toti-like virus (bootstrap value = 93). The last two *P. gracilis* EVEs are clustered within a well-supported clade of ant viruses such as *Camponotus yamaokai* virus and *Camponotus nipponicus* virus (bootstrap value = 99).

From the reconstructed RdRP Toti-Chryso phylogeny, *C. costatus* EVE25 is sister to the *L. boulardi* toti-like virus (bootstrap value = 85; Supplementary Figure S12).

#### ssRNA(RT) Viruses

##### Retroviruses (Metaviridae)

There were 93 ant EVEs similar to the ORF B (ORFs B) gene of *Trichoplusia ni* TED virus. This is an endogenous retrovirus found within the moth species, *T. ni*, and ORF B is a gene similar to the pol domain in retroviral genomes (Friesen and Nissen, 1990; Terzian et al., 2001). We could not reconstruct a phylogeny for these 93 EVEs because the ORF B gene has not been found in any other retroviruses to date.

#### ssDNA Viruses

##### Circoviridae

Replication-associated proteins (Rep) are responsible for genome replication within the viral Circoviridae clade (Dennis et al., 2018). Though weakly supported, in the Rep protein phylogeny, these two EVE hits are most closely related to the Dragonfly associated cyclovirus 2 (bootstrap value = 30; Supplementary Figure S13). *P. gracilis* EVE3 was previously discovered by Dennis et al. (2018) and conclusively, through targeted sequencing, shown to be an EVE in the *P. gracilis* genome.

##### Parvoviridae

Parvovirus genomes consist of a gene encoding a structural capsid protein (VP) and a non-structural protein, either Rep or NS (Bergoin and Tijssen, 2010; François et al., 2016). Many of the discovered viruses in the Parvoviridae clade have been found in vertebrate-hosts, however based on arthropod diversity, arthropod-host Parvoviridae viruses should vastly outnumber vertebrate viruses. In total, 12 ant EVEs are most similar to the Parvoviridae viral clade. The three EVE hits from the VP1 phylogeny all cluster together in a well-supported clade with Densovirus SC1065 (bootstrap value = 98; Supplementary Figure S14). The eight EVE hits from the NS1 phylogeny cluster into two different groups. Five of these EVEs cluster together into their own well-supported clade which is sister to a mosquito densovirus clade (bootstrap value = 98; Supplementary Figure S15). The other three EVEs cluster with the Lupine feces-associated densovirus in a moderately supported clade (bootstrap value = 65). Within the NS2 phylogeny, *Atta cephalotes* EVE5 clusters away from most of the insect densoviruses, but still clusters within a well-supported clade of several arthropod densoviruses (bootstrap = 98; Supplementary Figure S16).

#### dsDNA Viruses

##### Baculoviridae

*Per os* infectivity factor genes (PIF) and Baculovirus Repeated ORFs (Bro) are two common genes found within baculoviridae genomes which aid in host infection (Kang et al., 1999; Kikhno et al., 2002; Gauthier et al., 2015). A total of 17 ant EVEs are most similar to viruses from the Baculoviridae clade. The majority of these Baculoviridae EVE hits come from the *Harpegnathos saltator* genome (64.7% or 11 EVE hits), and all but one EVE is most similar to PIF fragments (PIF-1, PIF-2, and PIF-3). These EVEs which are similar to PIF fragments matched closely with *Apis mellifera* filamentous virus.

*Camponotus floridanus* EVE3 is most similar to a Bro-a fragment from *Mamestra brassicae* multiple nucleopolyhedrovirus. However, in the Bro-a phylogeny, this ant EVE did not fall anywhere within the nucleopolyhedrovirus lineage, which could suggest it is in its own viral genus within Baculoviridae (Supplementary Figure S17).

In the PIF-1 phylogeny, the eight EVE hits form a single ant-EVE specific distinct clade which is sister to *A. mellifera* filamentous virus (bootstrap value = 84; Supplementary Figure S18). Similarly, in the PIF-2 phylogeny the seven ant EVEs cluster together in a distinct clade with *A. mellifera* filamentous virus as the closest relative (bootstrap value = 83; Supplementary Figure S19). In addition, in the PIF-3 phylogeny the one EVE (*H. saltator* EVE1) clusters with *A. mellifera* filamentous virus (bootstrap value = 99; Supplementary Figure S20).

##### Poxviridae

There are a total of three ant EVEs which are most similar to the Poxviridae protein fragments Tryptophan repeat gene and RNA polymerase RPO147 (Theze et al., 2013; Mitsuhashi et al., 2014). The two EVEs within the tryptophan repeat gene phylogeny are found in separate clades within different lineages of entomopoxvirus (bootstrap value = 41; Supplementary Figure S21) and the *S. invicta* EVE1 clusters with the *Mythimna separata* entomopoxvirus (bootstrap value = 27). *L. humile* EVE1 falls within the RNA polymerase RPO147 clade, however it did not fall near any of the exogenous Entomopoxvirus viruses within this phylogeny (Supplementary Figure S22).

##### Polydnaviridae

Three ant EVEs are most similar to the *Cotesia congregata* bracovirus within the Polydnaviridae viral clade from the protein Pox A32 fragment (Espagne et al., 2004). In this phylogeny, all three EVEs fall into a clade with the *C. congregata* virus (bootstrap value = 100; Supplementary Figure S23).

### Stop Codon Analysis

Of the 238 EVE hits of the 434 ant EVEs we recovered do not contain random stop codons (Supplementary Table S4). Sixteen of these EVEs without nonsense mutations are comparable in length to the viral proteins to which they are most similar (Table 4). Therefore, these hits are considered potentially functional or recently acquired.

**Table 4:** Potentially functional stop codons.

EVE	Similar virus	Similar protein	EVE	Protein
			length (AA)	length (AA)
*Cyphomyrmex costatus* EVE18	Wuchang cockroach virus 3	Glycoprotein	586	659
*Linepithema humile* EVE6	Hubei narna-like virus 19	RdRP	750	737
*Monomorium pharaonis* EVE2	Densovirus SC1065	VP1	215	288
*Pogonomyrmex barbatus* EVE34	Wuhan mosquito virus 8	Glycoprotein	582	653
*Pseudomyrmex gracilis* EVE3	Cyclovirus PK5034	Rep-associated	236	277
*Pseudomyrmex gracilis* EVE4	Densovirus SC1065	VP1	281	288
*Pseudomyrmex gracilis* EVE41	Wuchang cockroach virus 3	Glycoprotein	641	659
*Pseudomyrmex gracilis* EVE45	Wuhan insect virus 16	Nucleoprotein	294	327
*Pseudomyrmex gracilis* EVE48	Wuhan mosquito virus 8	Glycoprotein	624	653
*Solenopsis invicta* EVE17	Wuhan mosquito virus 8	Glycoprotein	582	653
*Trachymyrmex cornetzi* EVE19	Wuchang cockroach virus 3	Glycoprotein	605	659
*Trachymyrmex septentrionalis* EVE19	Wuhan mosquito virus 8	Glycoprotein	612	653
*Vollenhovia emeryi* EVE10	Hubei partiti-like virus 11	Capsid	412	475
*Vollenhovia emeryi* EVE17	Wuhan insect virus 16	Nucleoprotein	327	327
*Vollenhovia emeryi* EVE19	Wuhan insect virus 16	Nucleoprotein	326	327
*Wasmannia auropunctata* EVE12	Wuhan mosquito virus 8	Glycoprotein	595	653


*Summary table of EVEs which did not contain stop codons and were similar in size to their most analogous protein. This table contains information on the EVE, the most similar virus to that EVE, the most similar protein to that EVE, the length of the EVE protein hit in amino acids, and the length of the most similar protein in amino acids.*

### EVE-Host Evolutionary Association Analyses

**FIGURE 3:**
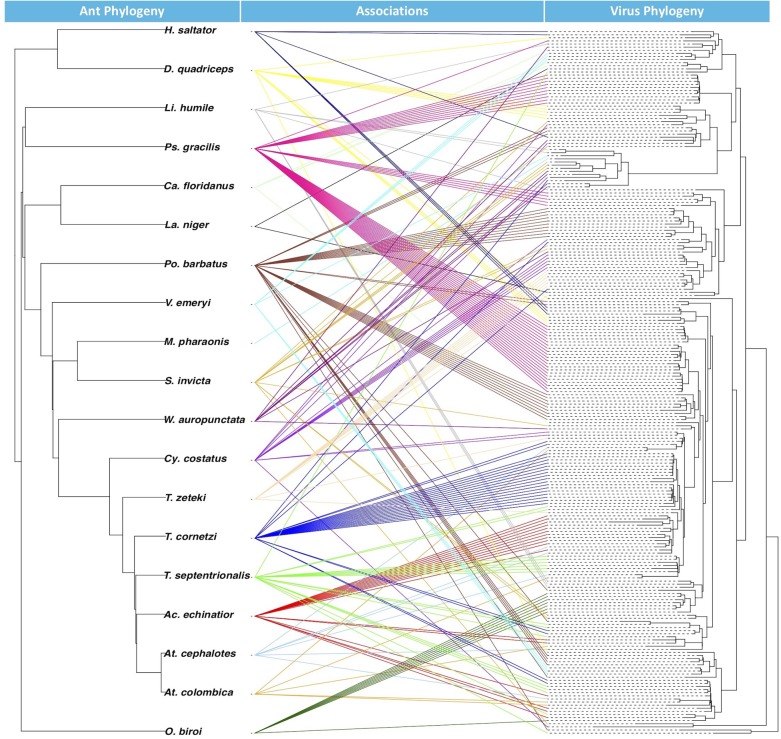
Tanglegram with ant phylogeny on left and glycoprotein Mono-Chu viral phylogeny on right. The exogenous viruses included in the viral phylogeny were left out because their hosts are not included in the ant phylogeny. Colors of the association are randomly prescribed for each of the ant species.

From the BaTS analysis, the association index ratio for the glycoprotein Mono-Chu EVE phylogeny in relation to the ant host phylogeny at the level of ant species was 0.263 with a significant *p*-Value of <0.001 and at the level of ant subfamily with a AI ratio of 0.090 and a significant *p*-Value of <0.001. From the JANE analysis of EVE-ant species co-divergence, we found that there were 26–30 co-divergence events, 65–68 host switching events, 131–132 duplication events 10–17 extinction events, and 278 total cost (combination of all non-co-divergence events) with a significant *p*-Value for the number of costs at <0.01. The tanglegram visualization illustrates the associations between ant phylogeny and the ant EVE phylogeny (Figure 3).

## Discussion

### EVE Diversity

This study has greatly expanded our knowledge of EVEs found within ants. EVE hits from ant genomes are derived from a strikingly diverse set of viral lineages both from RNA and DNA viruses. Overall, our phylogenetic analysis found that ant EVEs tend to group into distinct, well-supported clusters from more than 12 viral lineages. There are major differences in abundance in EVEs across ant genomes; *Lasius niger* has the fewest with only two EVEs whereas *C. costatus* has the largest number with 68 EVEs. This could reflect biologically distinct rates of endogenization by viruses into certain ant genomes. However, differences in genome sequencing and assembly quality may also contribute to this difference, as this might affect the number of EVEs one is able to detect in the genome. For example, when using long-read assembly for the *Aedes aegypti* genome, Whitfield et al. (2017) were able to discover a large and diverse number of EVEs. The different ant genomes vary considerably in their assembly statistics (Supplementary Table S3).

Statistically, the number of EVE hits per ant genome are not significantly correlated with any of the various factors relating to genome quality (genome length, scaffold statistics, contig statistics) (Figure 1 and Table 2). In addition, based on the synteny analysis, close to 80% of the scaffolds these EVE hits were found on were over 30,000 bp long and around 70% contained annotated host genes. This suggests that the quality of the genomes was not biasing these analyses in a substantial manner. However, even though no genome quality factors were significantly correlated, several were close to a significant correlation (contig N50 and genome length; Table 2). In addition, when we took the *L. niger* genome out of the Pearson’s product moment correlation analysis for these borderline significant tests, the correlation substantially decreased. This indicates the *L. niger* genome might be of lower quality than the other ant genomes and that genome quality does in fact constrain and potentially impact the abundance of EVE hits discovered.

In addition to the differences in EVEs per ant genome, there were marked differences in the number of EVEs per viral protein. It is plausible that certain viral types are better suited for attaining germline integration (Horie et al., 2010). For example, viruses which cause chronic infections in their host would most likely contain more EVEs than viruses solely causing acute infection (Holmes, 2011). Some viruses have been able to develop mechanisms to evade or inactivate the host’s cellular DNA repair machinery, which would allow them to integrate more successfully into the host genome. Several dsDNA viruses (adenoviruses) have been found to inactivate DNA repair proteins to evade excision (Weitzman et al., 2004;Lilley et al., 2007). Therefore, specific clades of endogenous viruses found in our study would potentially reach fixation more often in ant genomes depending on their strategy to evade excision.

Additionally, RNA viruses have higher rates of mutation and replication in comparison to DNA viruses, which lends itself to more frequent host switching and larger host species range (Sanjuan et al., 2010; Pompei et al., 2012). Host range of the viral clades can impact the distribution of EVEs across the phylogeny. For example, certain genes in betaretroviruses allow them to have broader host ranges than gammaretroviruses (Henzy and Johnson, 2013). Based on our results, viruses from the Mono-Chu RNA virus clade may have certain genes or strategies which enabled them to colonize and endogenize within every ant species analyzed.

### Understanding the EVE Phylogenies

Among many of the ssRNA viral protein phylogenies (Toti-Chryso, Narna-Levi, and Bunya-Arena) most ant EVEs cluster together and form monophyletic clades, suggesting they come from distinct and possibly ant-specific viral lineages. Interestingly many of these ant EVE-specific clades are most closely related to other insect or arthropod clades. Furthermore, almost all of the ssRNA viral lineages contained EVEs most similar to RdRP. This may be because RdRP is the only known conserved sequence domain across all RNA viruses and there are strong selection pressures acting on viral polymerases, leading to a high degree of conservation of such proteins (Hughes and Hughes, 2007; Shi et al., 2016). Qinvirus is a new ssRNA viral group discovered in 2016 and *C. floridanus* EVE7 is the first ant EVE from this viral lineage.

In addition to discovering many new ant EVEs, several of the EVEs previously discovered were reconfirmed in this study: *P. gracilis* EVE3 from the rep-association circoviridae phylogeny was previously found by Dennis et al. (2018). Three EVE hits (*Acromyrmex echinatior* EVE1,2 and *Monomorium pharaonis* EVE1) from the Parvoviridae NS1 phylogeny were previously found by François et al. (2016). This confirms that our methods were accurate in classifying EVEs.

*Camponotus floridanus* EVE3 in the Baculoviridae Bro-a protein phylogeny and *L. humile* EVE1 from the Baculoviridae RNA polymerase phylogeny did not fall out near any exogenous viruses. For this reason, these EVE hits may represent two new unknown viral clades. The three EVEs which were most similar to Polydnaviridae Pox A32 proteins are inferred to be most similar to the *C. congregata* virus, an endogenous wasp virus. This provides some evidence that these three lineages of ants could have been parasitized at some point over evolutionary history by a wasp species carrying this *C. Congregata* virus or alternatively that this virus has the potential to infect a diversity of Hymenoptera (ants, bees, and wasps). Similarly, over evolutionary history *H. saltator* ants could have been infected (and their genome endogenized) with an ancestor of the *A. mellifera* filamentous virus. This may be the reason that *H. saltator* genome contains several EVE protein fragments for PIF-1, PIF-2, and PIF-3; all proteins found within the *A. mellifera* filamentous virus.

One potential reason why there were so few retroviral EVEs discovered and so many RNA viruses discovered is an issue of sampling bias. There has been much more effort put into discovering RNA viruses within insects due to their impact on human populations (Dengue Virus, West Nile Virus, Zika Virus, and Yellow Fever Virus). Since, this pipeline can only identify the viruses available in the databases, a wide diversity and abundance of insect-specific RNA viruses were detected.

Retroviral ORF B EVE hits constituted around 21% of the total EVE hits for all ant genomes. Retroviral EVEs make up a large proportion of hits because endogenization into the host genome forms part of their replication cycle. However, it is still surprising that an even more diverse group of endogenous retroviruses were not found. One of the reasons we suspect that solely ORF B analogs were discovered for retroviruses is because there are only thirteen described insect-specific retroviruses. One of these thirteen described retroviruses is *T. ni* TED virus, which contains this ORF B gene (Terzian et al., 2001). These ORF B ant EVE hits are most similar to the moth endogenous retrovirus *T. ni* TED virus, which functions as a retrotransposon in the moth genome. This suggests that these EVEs could be classified as retrotransposons and should be further analyzed to better understand their evolutionary role in the ant genomes.

Many of the ant EVEs discovered in this study tend to cluster together in clades by ant species. Multiple EVEs from the same ant species form distinct clades seven times in the Mono-Chu glycoprotein phylogeny, twice in Toti-Chryso coat phylogeny, twice in the Bunya-Arena nucleoprotein phylogeny, once in the Partiti-Picobirna capsid phylogeny, and once in each of the Baculoviridae PIF-1 and PIF-2 phylogenies. This suggests that EVEs have often been generated either by multiple viral integration events or by one integration event and multiple duplication events. In addition, it implies that ant species are either more prone to persistent infection by viruses in certain lineages or that this viral sequence was repeatedly conserved in these specific ant genome over evolutionary time.

### Mono-Chu Glycoprotein Analyses

We chose to focus much of the analyses on the Mono-Chu glycoprotein phylogeny as it was the only group where there were EVEs in all 19 ant genomes, therefore aiding in a more comparative analysis of the EVEs. One potential reason that glycoproteins constitute over half of the EVEs identified may be because viral glycoproteins are extremely important in viral infection and immunity. Glycoproteins often play a critical function in viral infection by identifying and binding to receptor sites on the host’s membrane (Banerjee and Mukhopadhyay, 2016).

The 224 EVEs, which make up Clades 2–20 in the Mono-Chu glycoprotein phylogeny, all fall within a distinct ant-specific lineage not closely related to any exogenous viruses (bootstrap value = 64; Figure 2B). The considerable divergence between these ant EVEs and any other exogenous virus suggests that this lineage most likely represents a previously undiscovered clade of viruses. Since so few viruses currently infecting ants (and insects) have been identified, the additional resolution of this phylogeny with newly discovered viruses will help us understand if this Mono-Chu viral lineage consists of solely ant- or insect- specific viruses.

Since this Mono-Chu glycoprotein phylogeny contains so many ant EVEs, several interesting inferences can be made. Clade 14 consists solely of 48 fungus-growing ant EVEs. This clustering could be potentially due to infection with a virus specific to fungus-growing ants or alternatively, a prior infection in the ancestor of all fungus-growing ants. Clade 3 exhibits extremely long branch lengths compared to the other clades within this phylogeny. This implies that the infecting viruses endogenized fragments into the ant species in this clade longer ago in evolutionary time, giving this clade time to diverge from the rest of the viral fragments in this phylogeny, although there could also be faster rates of molecular evolution in this virus. As in many of the other inferred phylogenies, EVEs from certain ant species tend to clump together into distinct clades. This happens frequently in the Mono-Chu glycoprotein phylogeny. For example, *P. gracilis* EVEs cluster together into three distinct clades (Clades 4, 13, and 17). This suggests that *P. gracilis* might be more prone to viral infection by viruses from the Mono-Chu lineage than other ant species.

Examination of EVE-ant association reveals that though host switching is most likely common among these viruses, EVEs can mirror their ant hosts over evolutionary time within the Mono-Chu lineage. This is supported by the AI ratios, which found that the EVE phylogeny exhibited significant clustering (*p* < 0.001) by host taxonomy for both ant species and ant subfamily. The ant subfamily (0.090) exhibited much stronger structuring than for the ant species (0.263), however both of these values are much closer to 0 than to 1, suggesting that the ant species are strongly structuring the EVE phylogenetic relationships. The co-phylogenetic analysis was performed at the ant species level, and found significantly more EVE-ant host co-divergence than solely by chance (*p* < 0.01). Overall this implies a long-term association of Mono-Chu viruses and their ant hosts. However, host-switching of these EVEs seems to also be very frequent throughout their evolution, and even more common than co-divergence with ant species in the Mono-Chu viral glycoprotein lineage. Therefore, even though there looks to be a long-term association between viruses from the Mono-Chu lineage in ant genomes, cross-species host switching occurred commonly as the viruses/ants co-diverged. One can visually assess this association from the tanglegram, which illustrates topological incongruence that suggests host-switching, though the EVEs tend to clump by ant species implying potential for co-divergence (Figure 3).

### Functionality

Around half of the EVEs we found across the ant genomes included nonsense mutations from premature stop codons. These stop codons tend to accumulate over evolutionary time in parts of the genome which are not functional. However, 238 EVE hits (54.8% of all EVEs discovered) did not contain stop codons (Supplementary Table S4) and are considered intact ORFs. In addition, sixteen of these hits were roughly the same size as the viral proteins they were most similar to, which implies that these sequences are still potentially functional within the ant genome (Table 4). Finding these potentially functional ORFs could either mean that there was a recent origin of the EVE or that the fragment was conserved over evolutionary time. If it is the latter, then these EVEs could potentially serve a current function in the genome, such as an antiviral defense mechanism co-opted by the ant. The genomes of certain ant species might also be predisposed to accumulating more functional or non-functional EVEs depending on population demographics and exposure to specific viruses over time.

## Conclusion

Newly discovered EVEs were found within all ant genomes and are similar to a large diversity of viral lineages. Many of these viral lineages do not contain currently known exogenous viruses from ant hosts, although several are closely related to other insect and arthropod exogenous viruses. Certain ant genomes tend to contain more abundant EVEs within them. Many closely related EVEs tend to cluster by species, which suggests multiple integration or duplication events within ant species. In addition, through analysis of EVEs similar to viral glycoproteins, host switching appears to be common among EVEs found in ants, though many EVEs have long-term associations with ant species and ant subfamilies. Furthermore, the potential for functionality of several of these EVEs supports the idea that EVEs could be playing an important role in ant genomes.

## Author Contributions

PF and CM conceived, designed, and executed the study and revised the manuscript. PF analyzed the data and wrote the manuscript.

## Conflict of Interest Statement

The authors declare that the research was conducted in the absence of any commercial or financial relationships that could be construed as a potential conflict of interest.

## Footnotes

1 https://www.ncbi.nlm.nih.gov/; accessed 1/1/2018
