# Supplementary material for: Assessing the Diversity of Endogenous Viruses Throughout Ant Genomes
Source: Front Microbiol. 2019 May 22;10:1139. doi: 10.3389/fmicb.2019.01139 (PMC6540820; doi:10.3389/fmicb.2019.01139)
Supplement: Supplementary file 4 [file Table_4.DOCX]

***Supplementary Material***

**Supplemental Figure (SF)**

**Figure S1-23**: Phylogenies obtained from maximum likelihood analysis of the multiple amino acid alignment, including both ant EVE sequences and closely related exogenous viruses. ML nonparametric bootstrap values <70 (1000 replicates) are indicated at each node. The tips are colored by associated host.

**Fig. S1.** Bunya-Arena Nucleoprotein Phylogeny. Best fit protein substitution model was VT + G + F.

**Fig. S2.** Bunya-Arena RNA-dependent RNA polymerase Phylogeny. Best fit protein substitution model was LG + I + G + F.

**Fig. S3.** Hepe-Virga RNA-dependent RNA polymerase Phylogeny. Best fit protein substitution model was LG + I + G + F.

**Fig. S4.** Glycoprotein Mono-Chu Phylogeny. Best fit protein substitution model was JTT + G + F.

**Fig. S5.** Mono-Chu Nucleoprotein Phylogeny. Best fit protein substitution model was LG + I + G + F.

**Fig. S6.** Mono-Chu RNA-dependent RNA polymerase Phylogeny. Best fit protein substitution model was LG + I + G + F.

**Fig. S7.** Narna-Levi RNA-dependent RNA polymerase Phylogeny. Best fit protein substitution model was LG + I + G + F.

**Fig. S8.** Partiti-Picobirna Capsid Protein Phylogeny. Best fit protein substitution model was LG + I + G + F.

**Fig. S9.** Partiti-Picobirna RNA-dependent RNA polymerase Phylogeny. Best fit protein substitution model was LG + G + F.

**Fig. S10.** Qinvirus RNA-dependent RNA polymerase Phylogeny. Best fit protein substitution model was LG + I + G + F.

**Fig. S11.** Toti-Chryso Coat Protein Phylogeny. Best fit protein substitution model was VT + G + F.

**Fig. S12.** Toti-Chryso RNA-dependent RNA polymerase Phylogeny. Best fit protein substitution model was LG + I + G.

**Fig. S13.** Circoviridae Replication-associated protein Phylogeny. Best fit protein substitution model was WAG + I + G + F.

**Fig. S14.** Parvoviridae VP1 Phylogeny. Best fit protein substitution model was WAG + G + F.

**Fig. S15.** Parvoviridae Non-Structural Protein 1 Phylogeny. Best fit protein substitution model was VT + I + G + F.

**Fig. S16.** Parvoviridae Non-Structural Protein 2 Phylogeny. Best fit protein substitution model was RTREV + G.

**Fig. S17.** Baculoviridae Bro-a Phylogeny. Best fit protein substitution model was LG + G + F.

**Fig. S18.** Baculoviridae PIF-1 Phylogeny. Best fit protein substitution model was VT + I + G.

**Fig. S19.** Baculoviridae PIF-2 Phylogeny. Best fit protein substitution model was BLOSUM62 + I + G + F.

**Fig. S20.** Baculoviridae PIF-3 Phylogeny. Best fit protein substitution model was FLU + I + G + F.

**Fig. S21.** Poxviridae Tryptophan protein Phylogeny. Best fit protein substitution model was CPREV + G + F.

**Fig. S22.** Poxviridae RNA polymerase Phylogeny. Best fit protein substitution model was LG + G + F.

**Fig. S23.** Polydnaviridae PoxA32 protein Phylogeny. Best fit protein substitution model was VT + G + F.

**Supplementary Tables**

**Table S1.** Excel file with information about each EVE identified including: EVE hit name, Ant Species which EVE hit was identified in, exact location in genome EVE hit was found, most closely related virus to EVE hit, most closely related virus family, viral protein information, viral protein accession number from NCBI, protein sequence of EVE hit, EVE hit length in amino acids, original query frame, % pairwise identity to protein, E-value, scaffold length which EVE hit was found on in nucleotide basepairs, number of host genes annotated on scaffold, and information on if the EVE hit was manually concatenated.

**Table S2. ﻿**Nucleotide sequences of all endogenous viral elements identified in this study.

**Table S3.** Excel file with information about both the clipped and pre-clipped genome. The column information includes: ant species, number of contigs, number of scaffolds, scaffold N50 (in mb), scaffold L50 (in mb), contig N50 (in kb), contig L50 (in kb), GC%, genome length (in mb), and number of EVE hits.

**Table S4.** Excel file with information on every EVE which did not contain a stop codon. The column information includes: ant EVE hit name, most similar virus, most similar protein, EVE hit length, most similar protein length, if this hit was potentially functional, the original query frame, protein accession number, EVE hit with scaffold identification, and protein sequence.
