# Supplementary material for: Assessing the Diversity of Endogenous Viruses Throughout Ant Genomes
Source: Front Microbiol. 2019 May 22;10:1139. doi: 10.3389/fmicb.2019.01139 (PMC6540820; doi:10.3389/fmicb.2019.01139)

Figure S1: Bunya-Arena Nucleoprotein Phylogeny

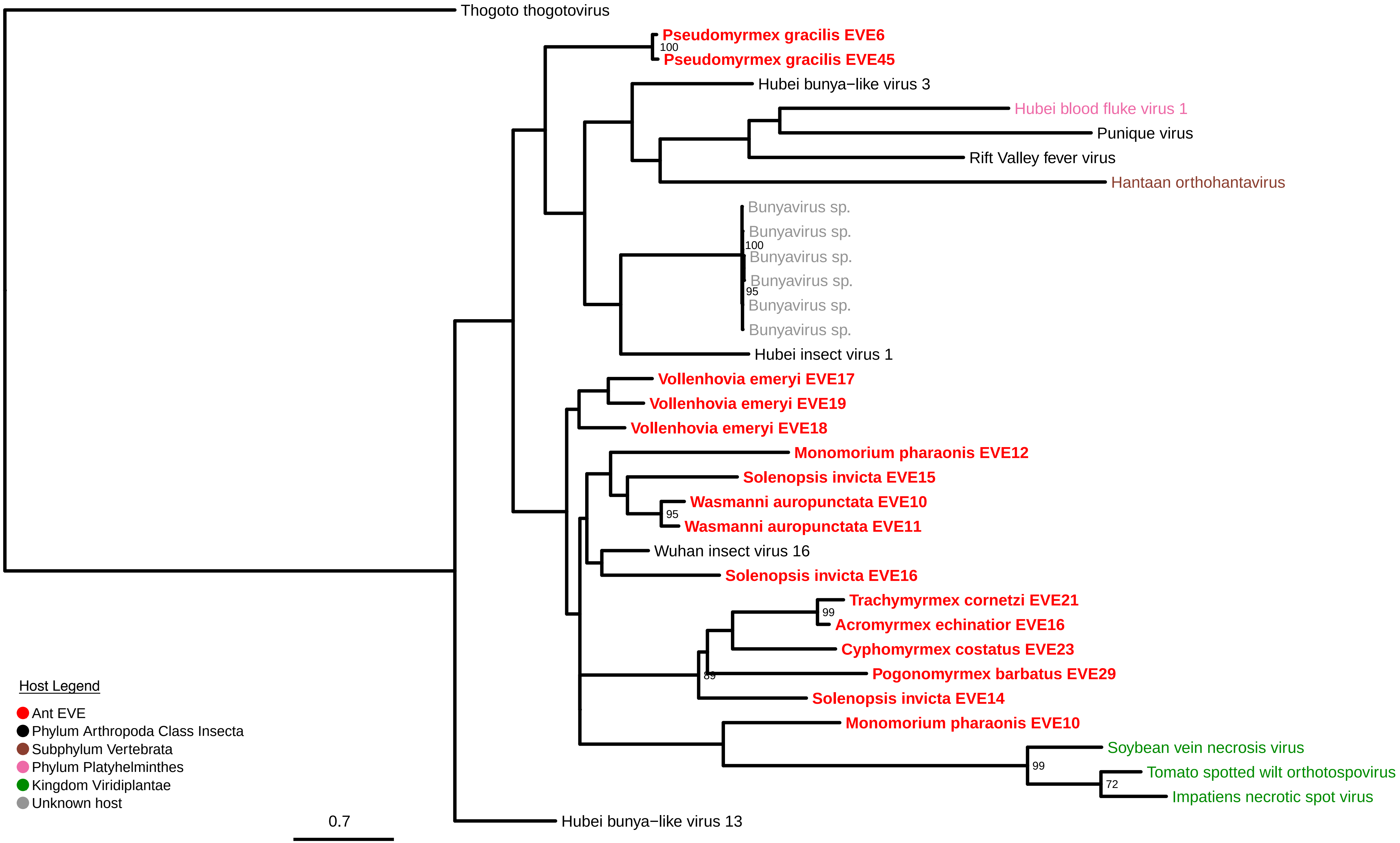

Figure S2: Bunya-Arena RNA-dependent RNA polymerase Phylogeny

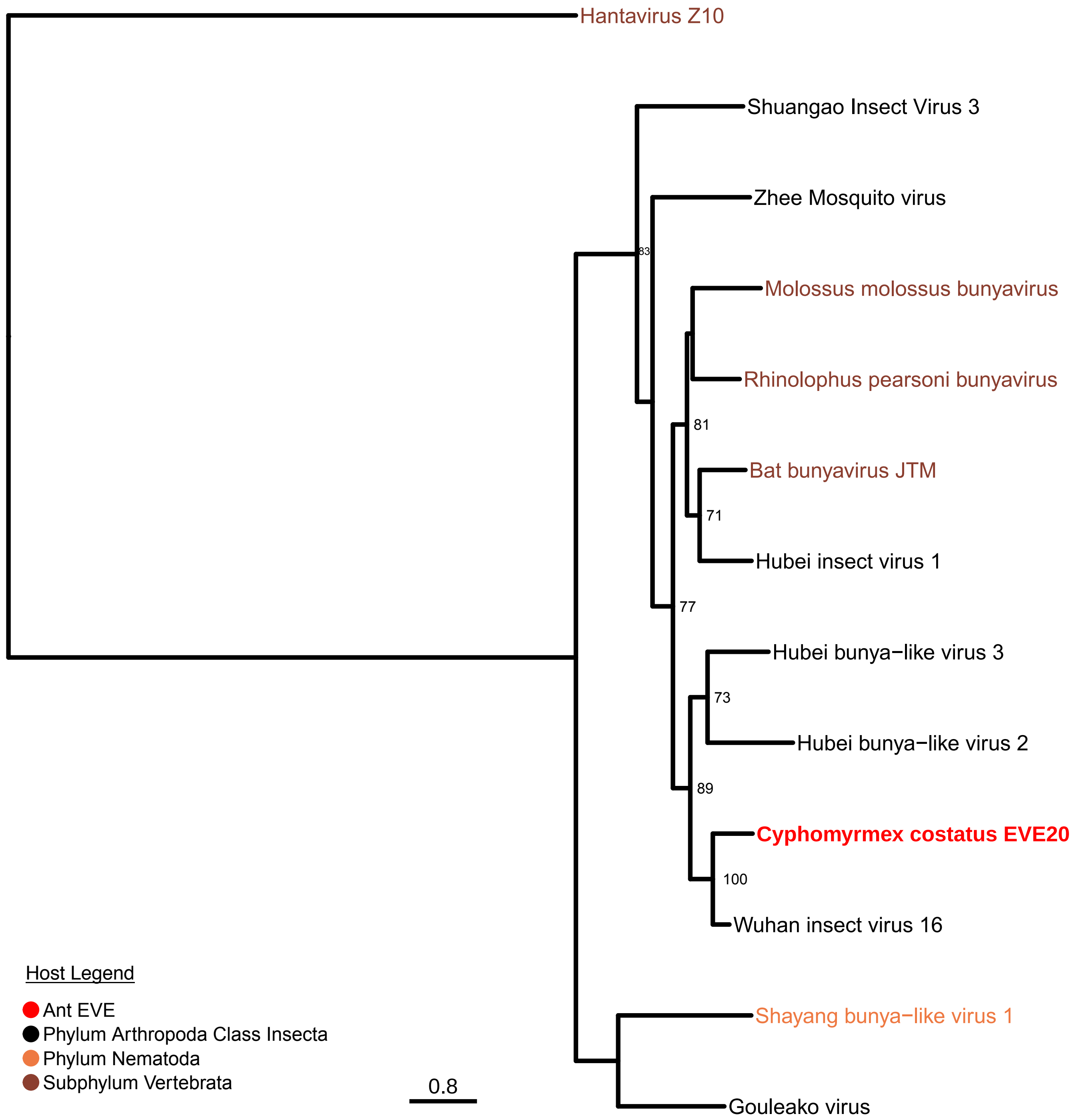

Figure S3: Hepe-Virga RNA-dependent RNA polymerase Phylogeny

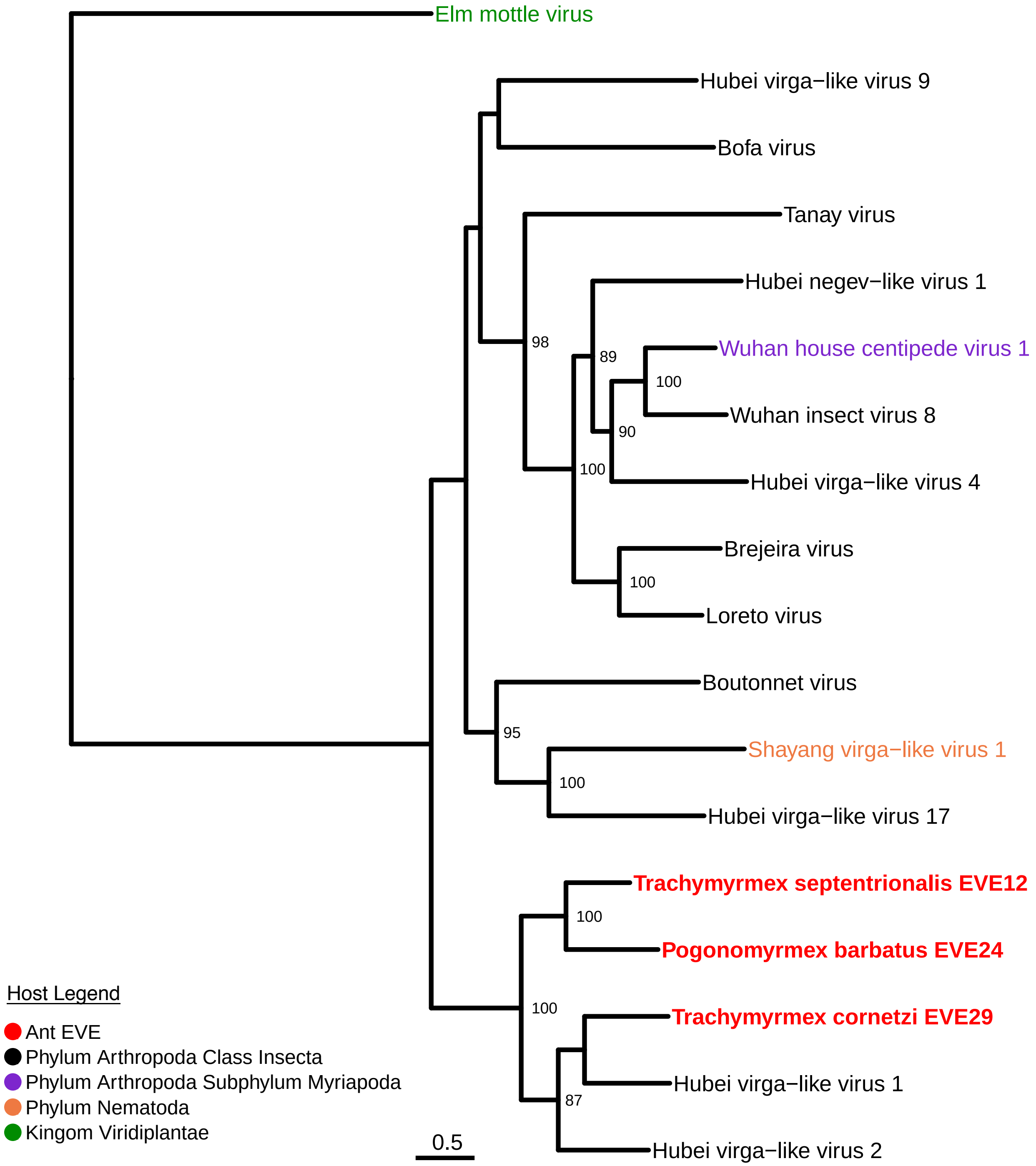

Figure S4: Mono-Chu Glycoprotein Phylogeny

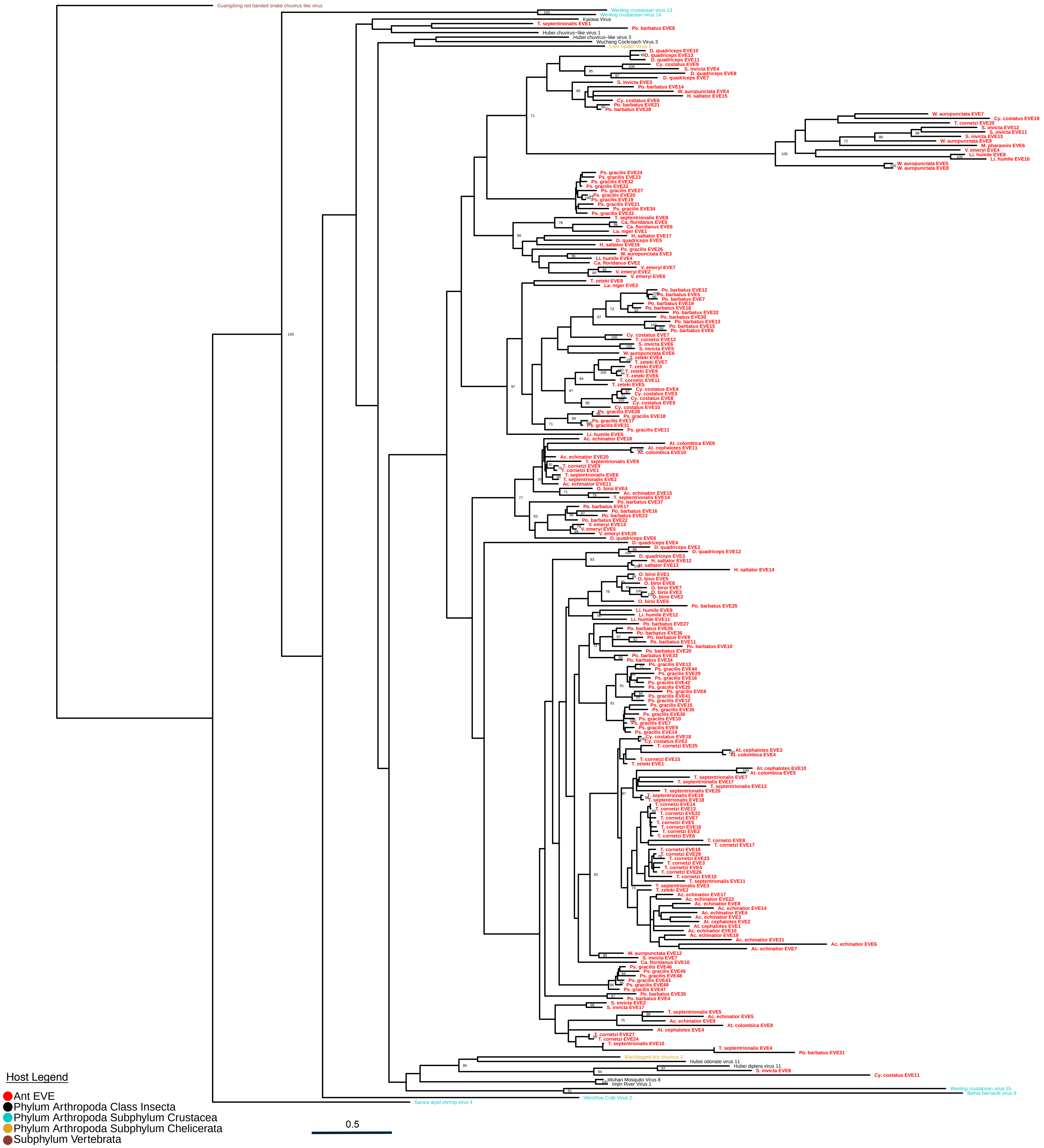

Figure S5: Mono-Chu Nucleoprotein Phylogeny

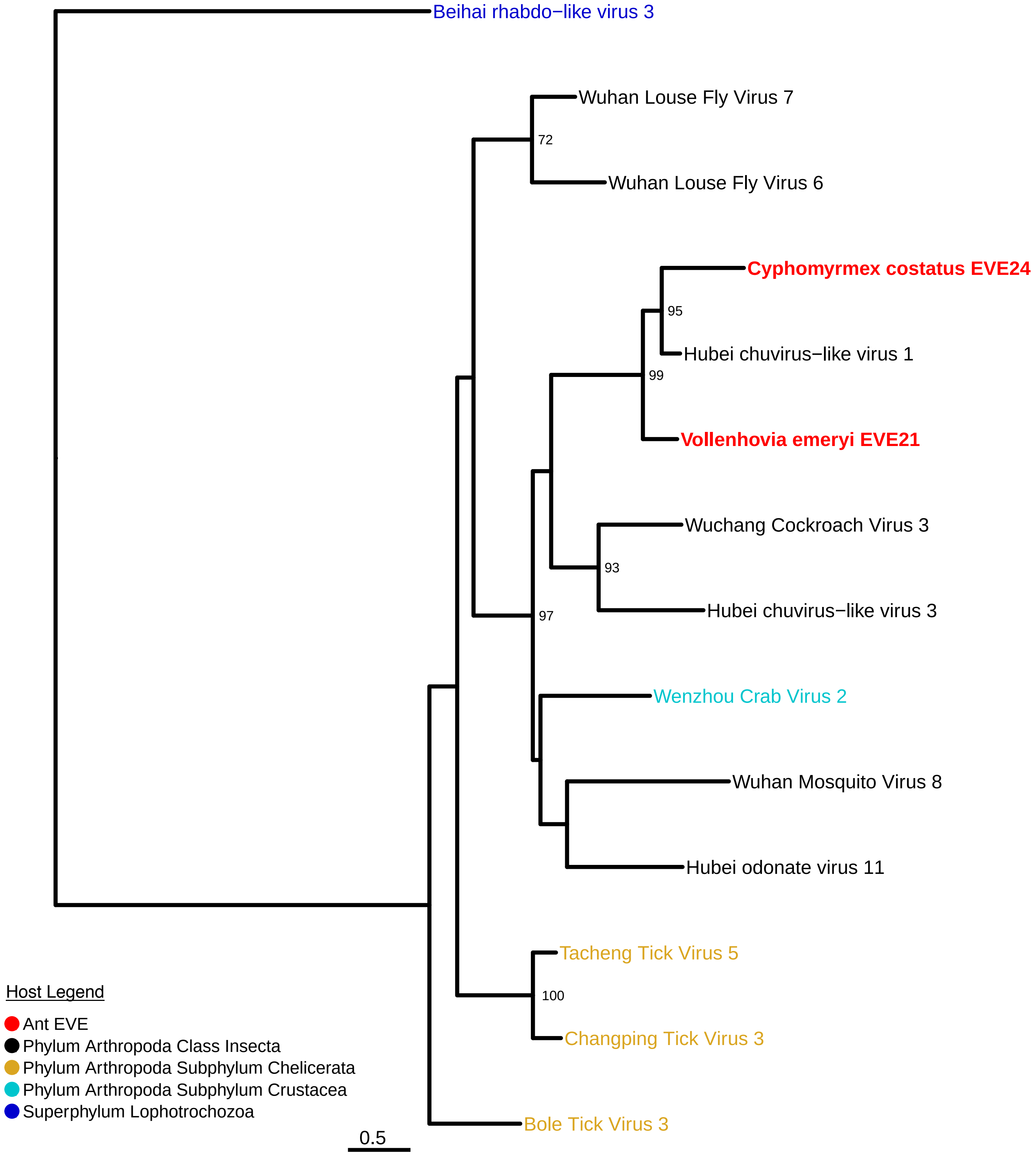

Figure S6: Mono-Chu RNA-dependent RNA polymerase Phylogeny

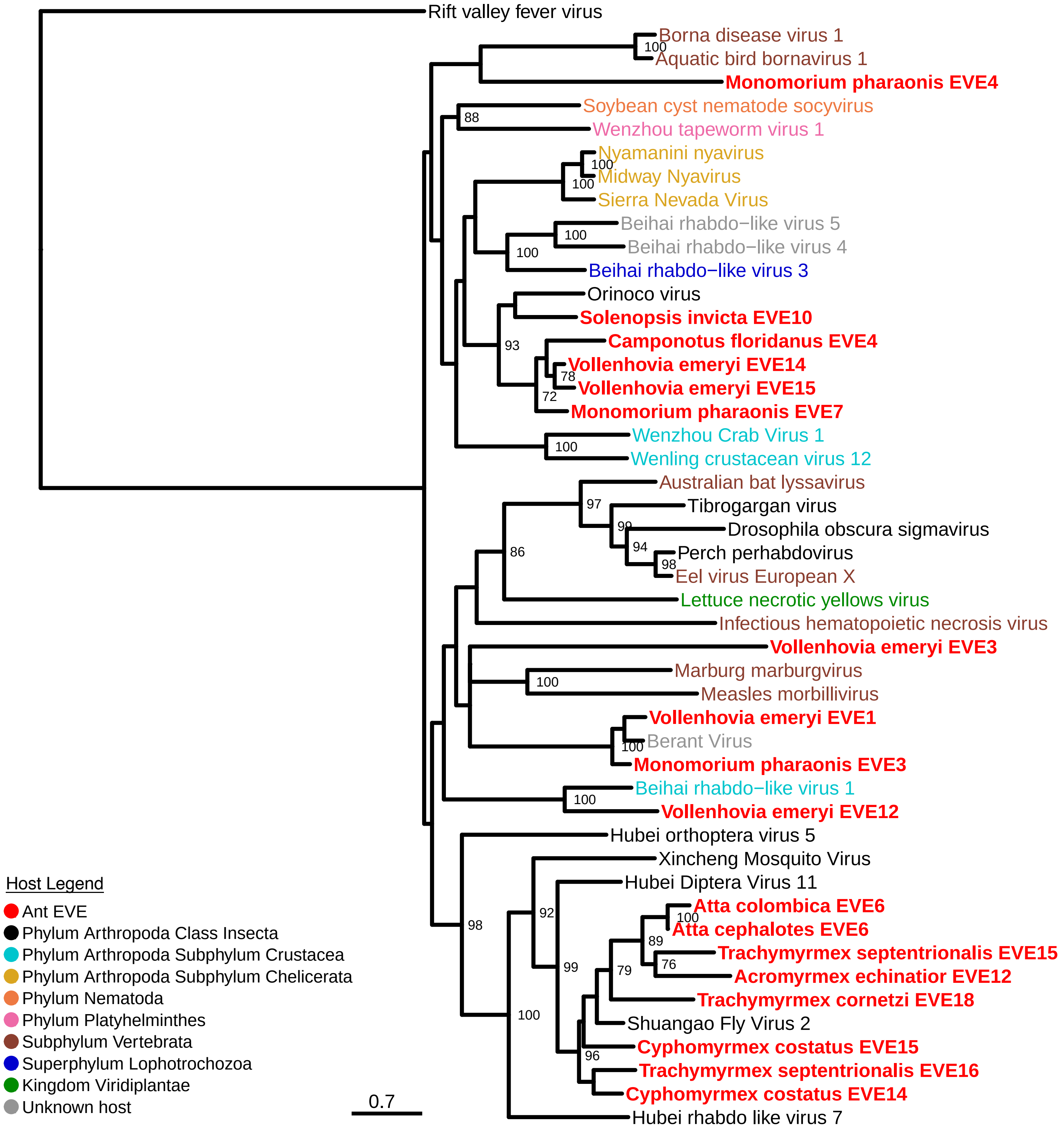

Figure S7: Narna-Levi RNA-dependent RNA polymerase Phylogeny

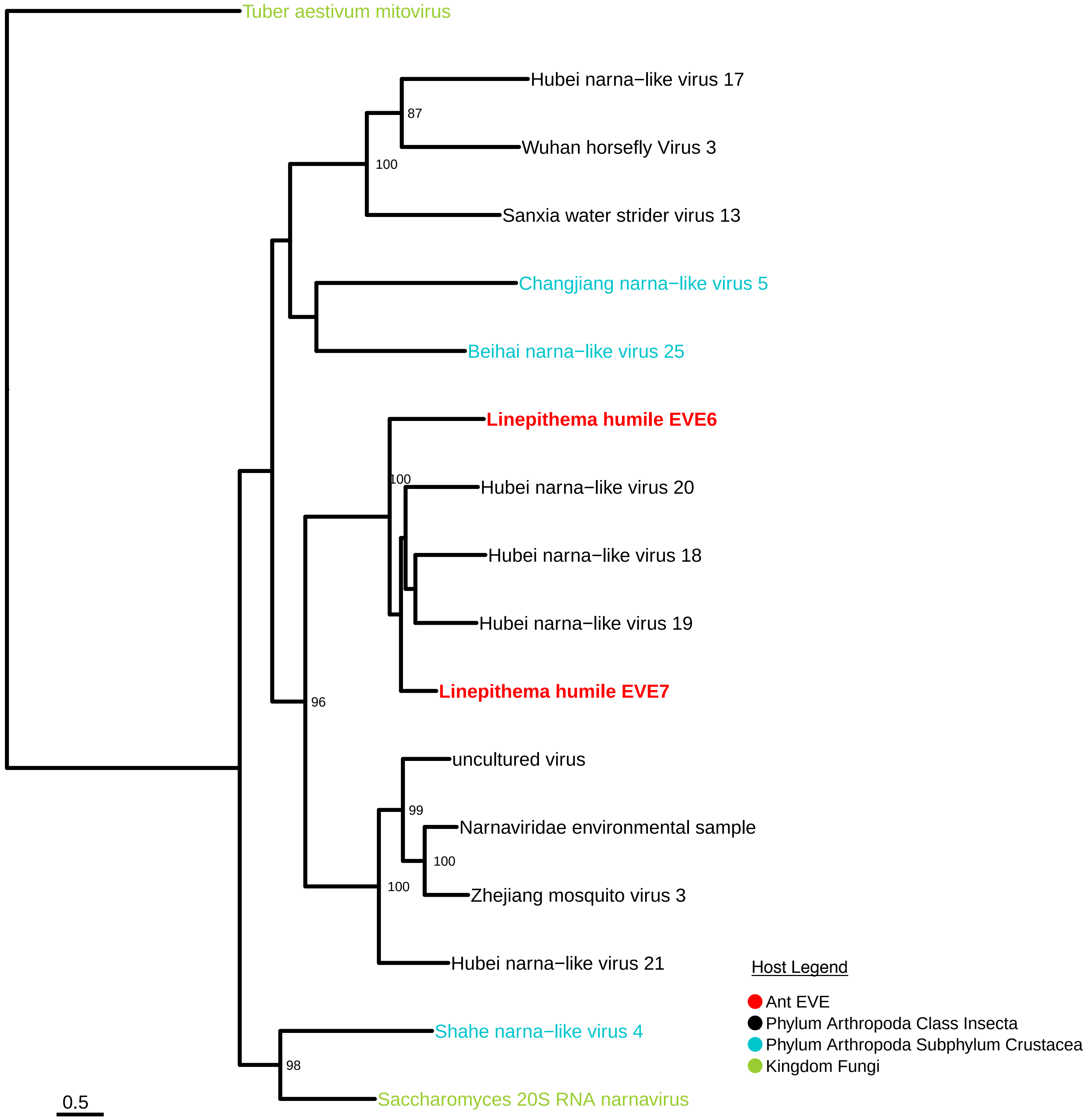

Figure S8: Partiti-Picobirna Capsid Protein Phylogeny

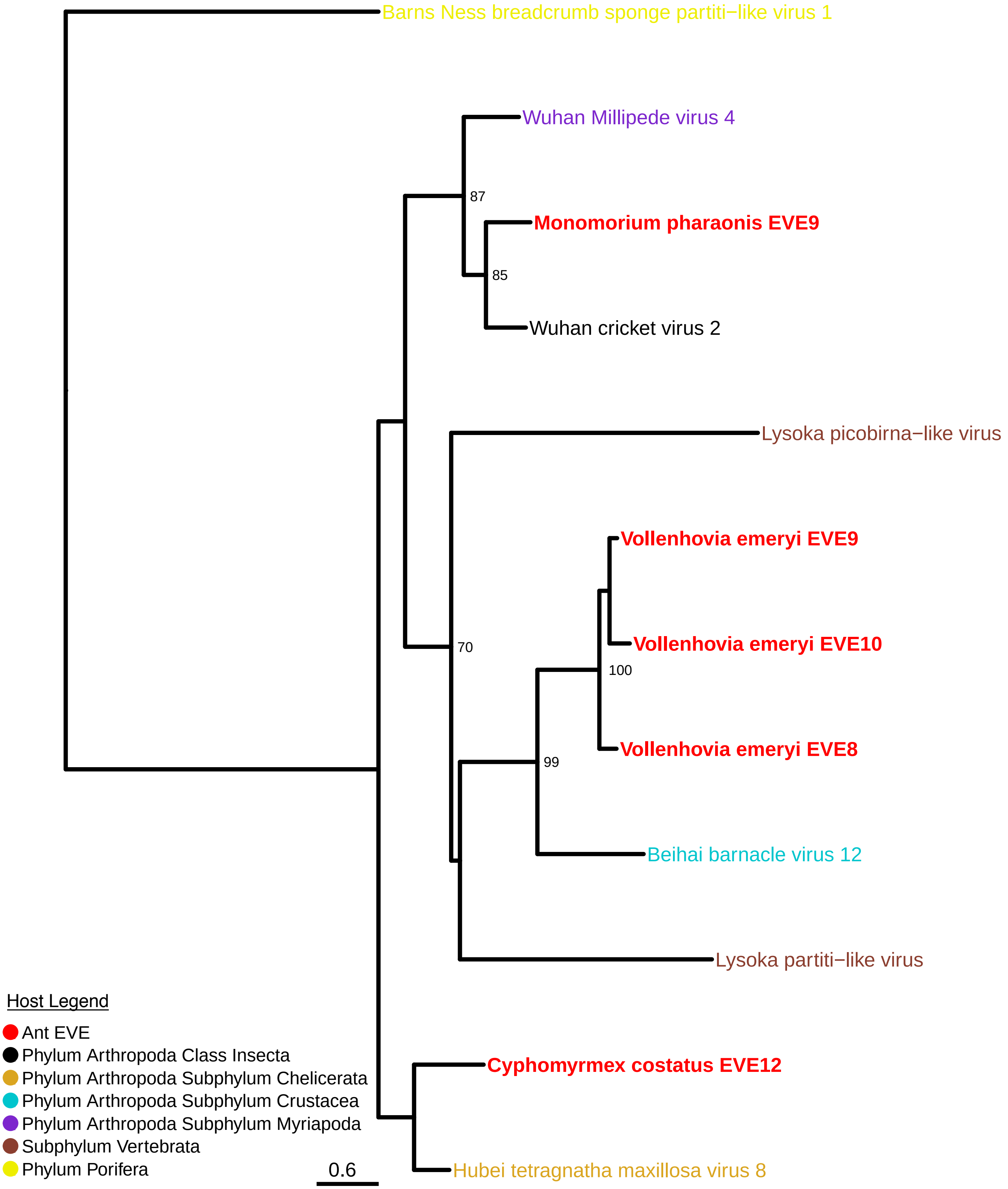

Figure S9: Partiti-Picobirna RNA-dependent RNA polymerase Phylogeny

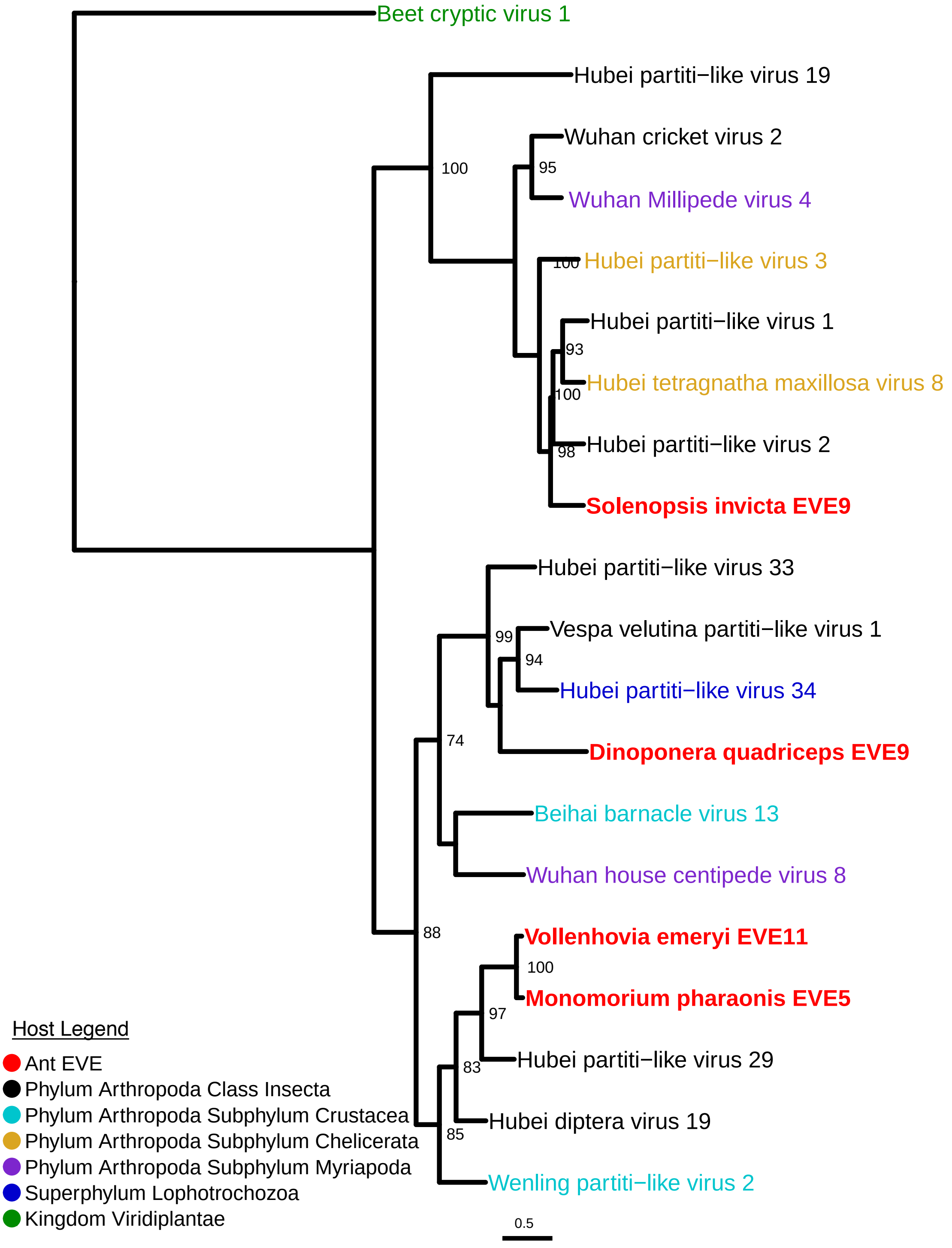

Figure S10: Qinvirus RNA-dependent RNA polymerase Phylogeny

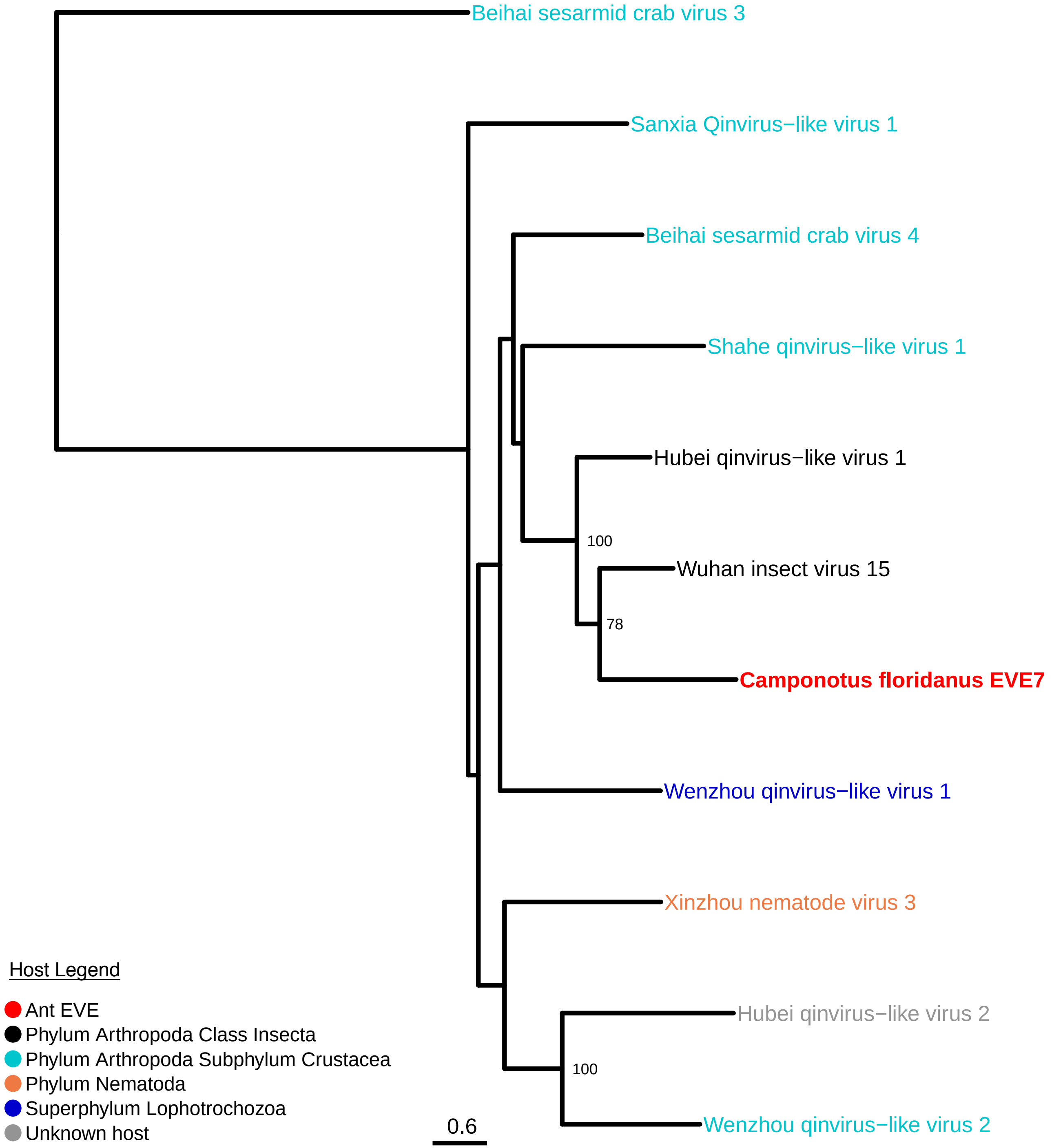

Figure S11: Toti-Chryso Coat Protein Phylogeny

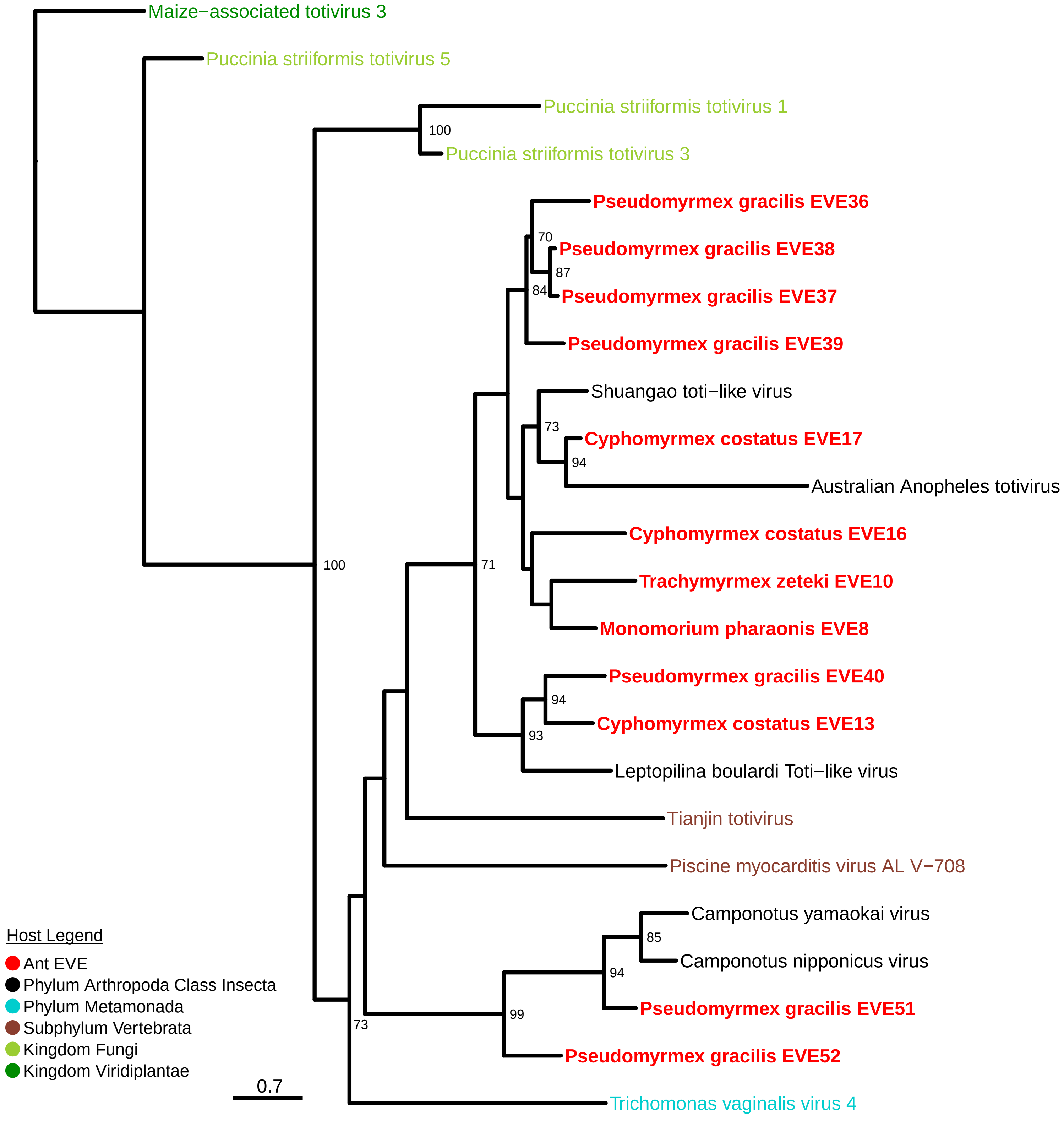

Figure S12: Toti-Chryso RNA-dependent RNA polymerase Phylogeny

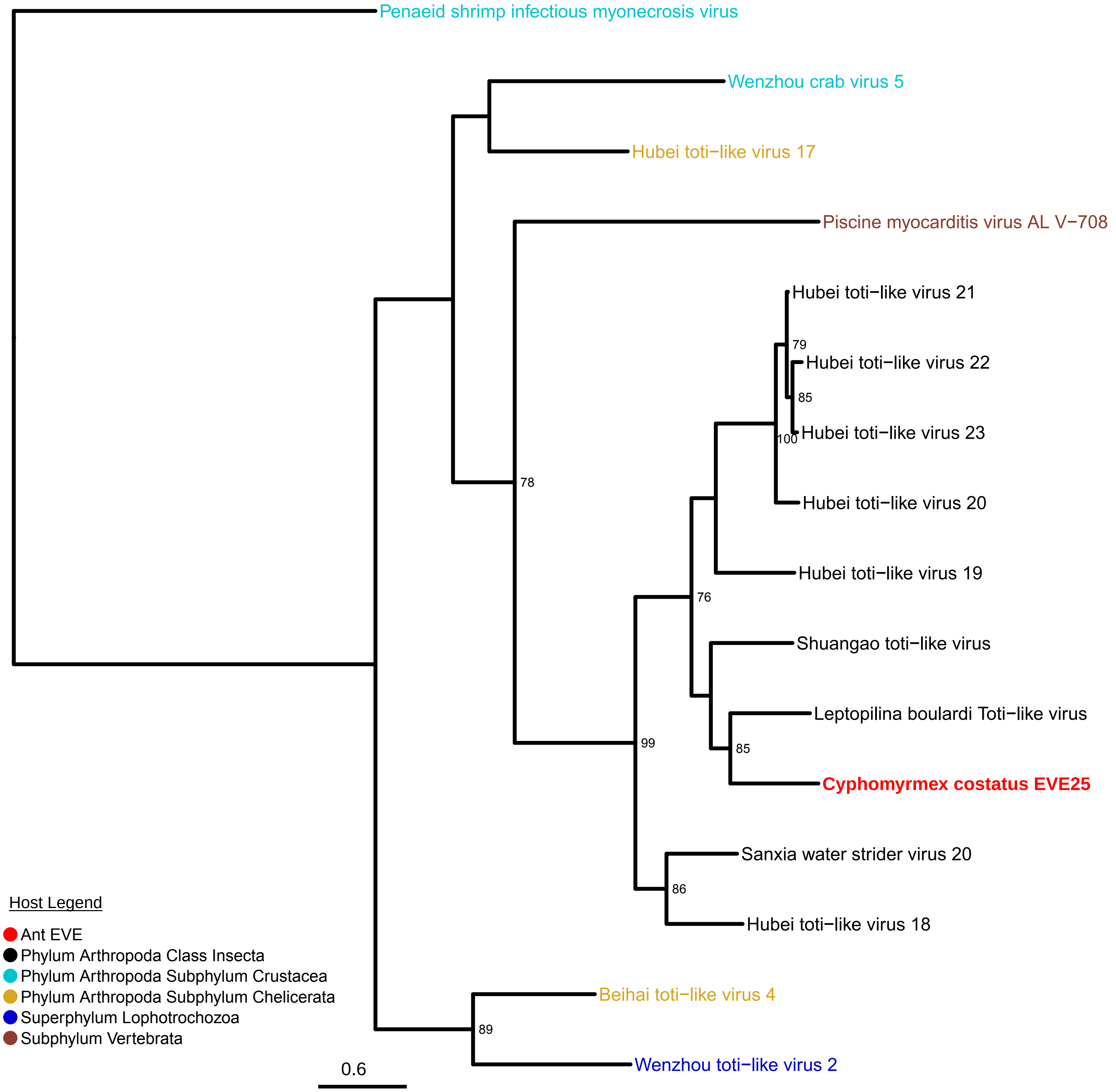

Figure S13: Circoviridae Replication-associated protein Phylogeny

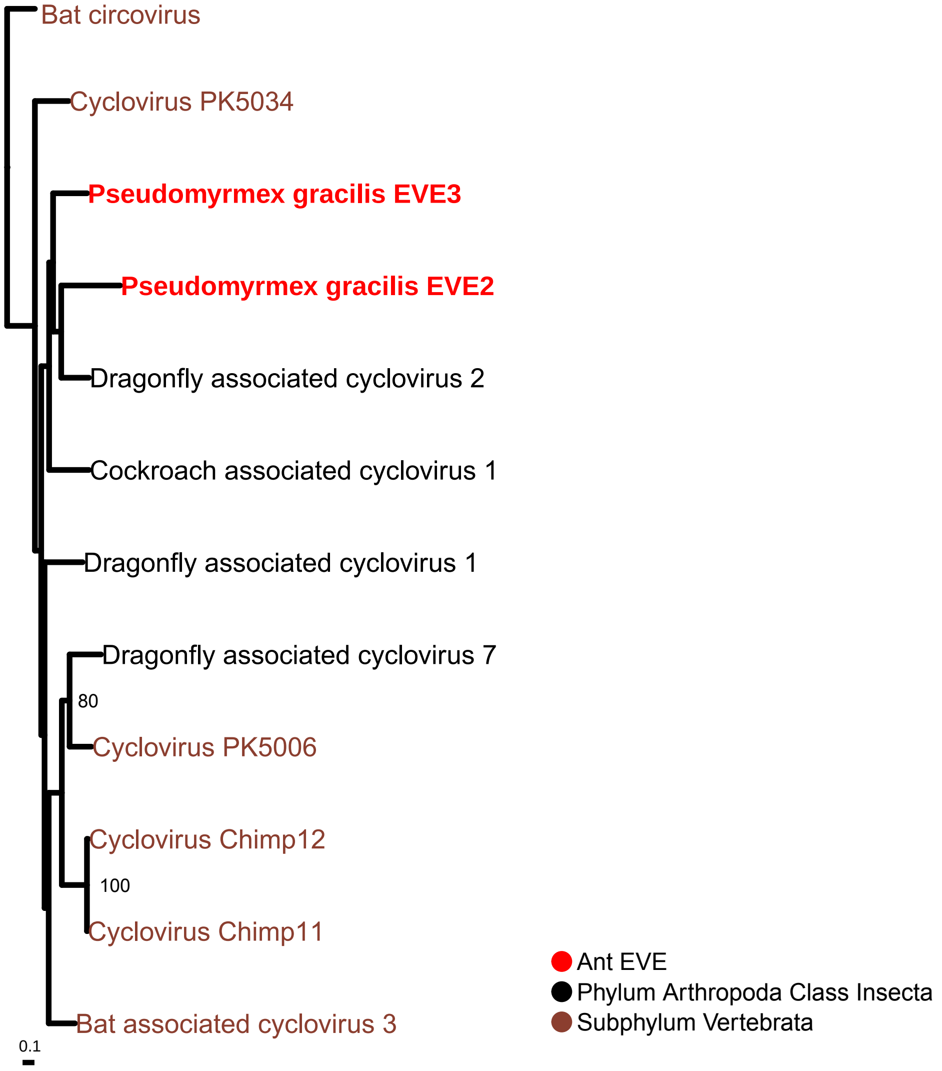

Figure S14: Parvoviridae VP1 Phylogeny

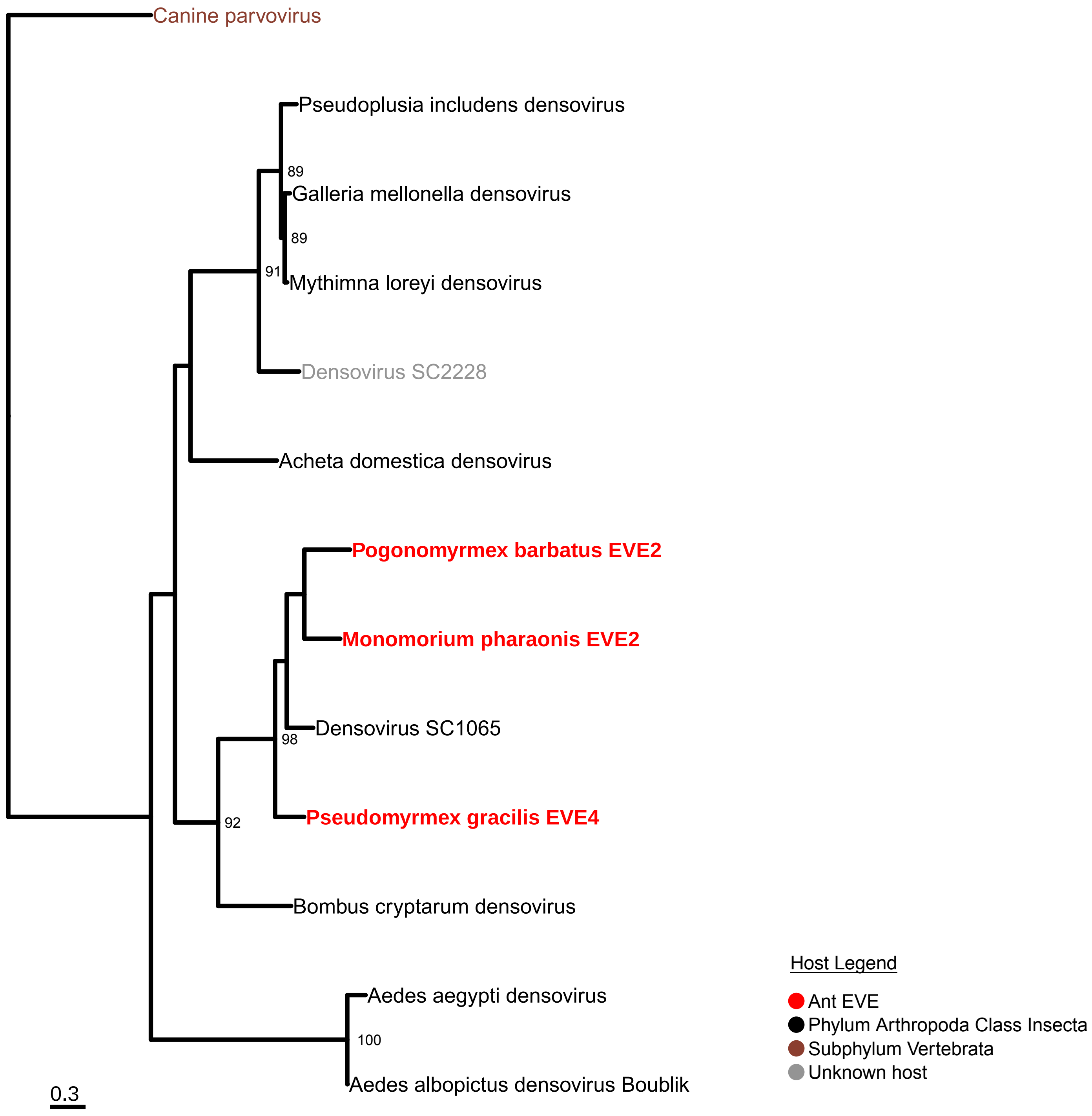

Figure S15: Parvoviridae Non-Structural Protein 1 Phylogeny

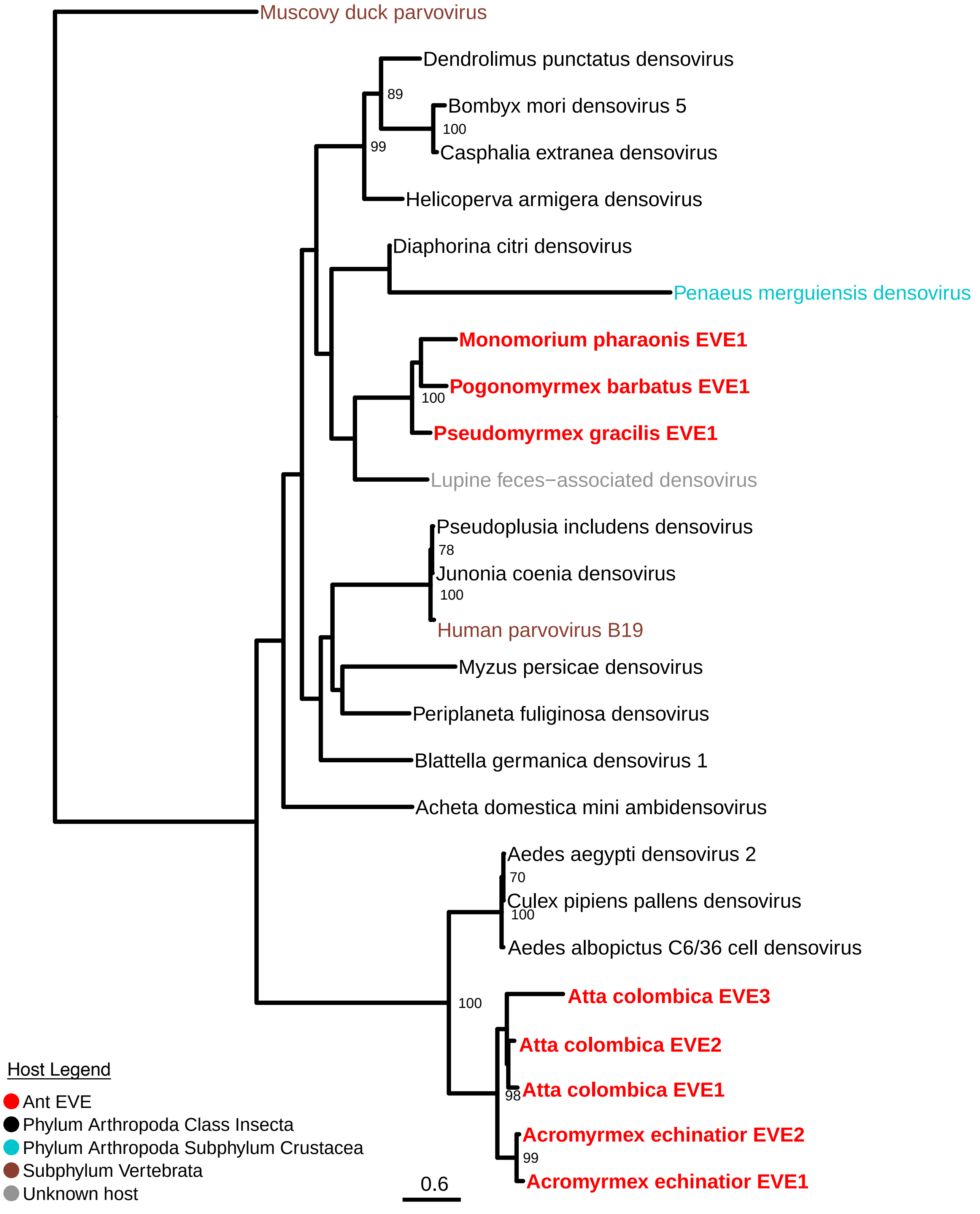

Figure S16: Parvoviridae Non-Structural Protein 2 Phylogeny

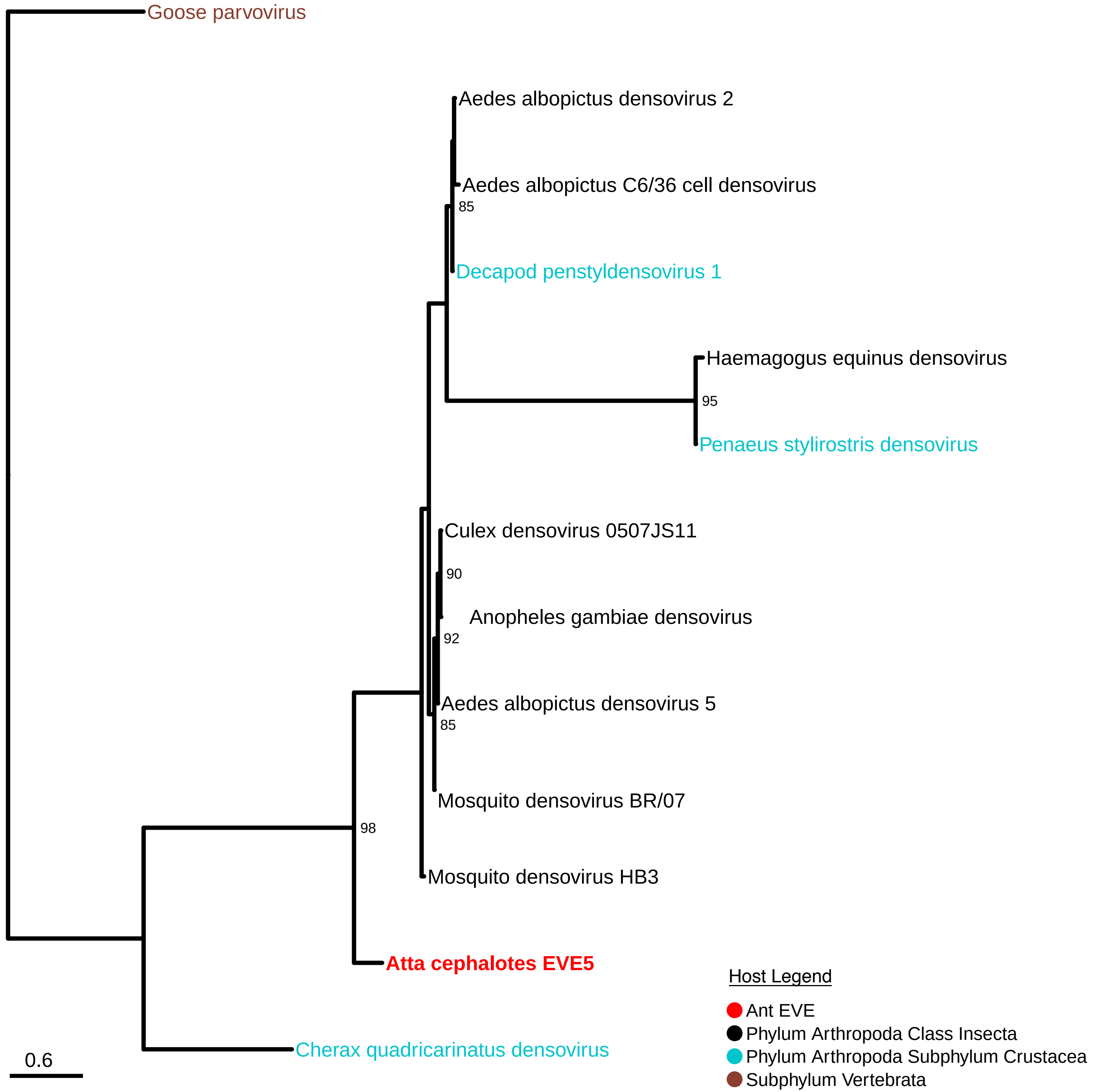

Figure S17: Baculoviridae Bro-a Phylogeny

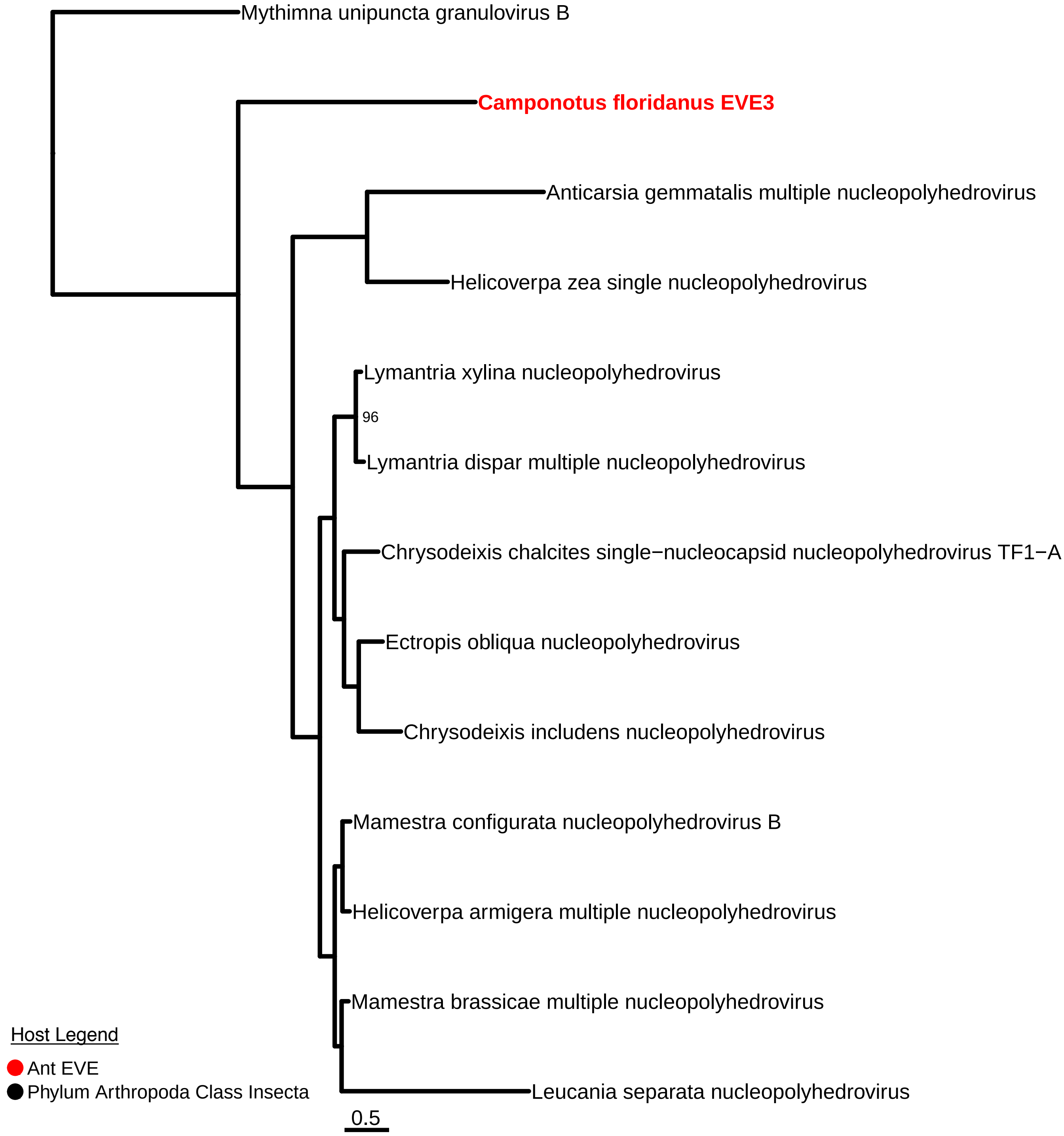

SF-18: Baculoviridae PIF-1 Phylogeny

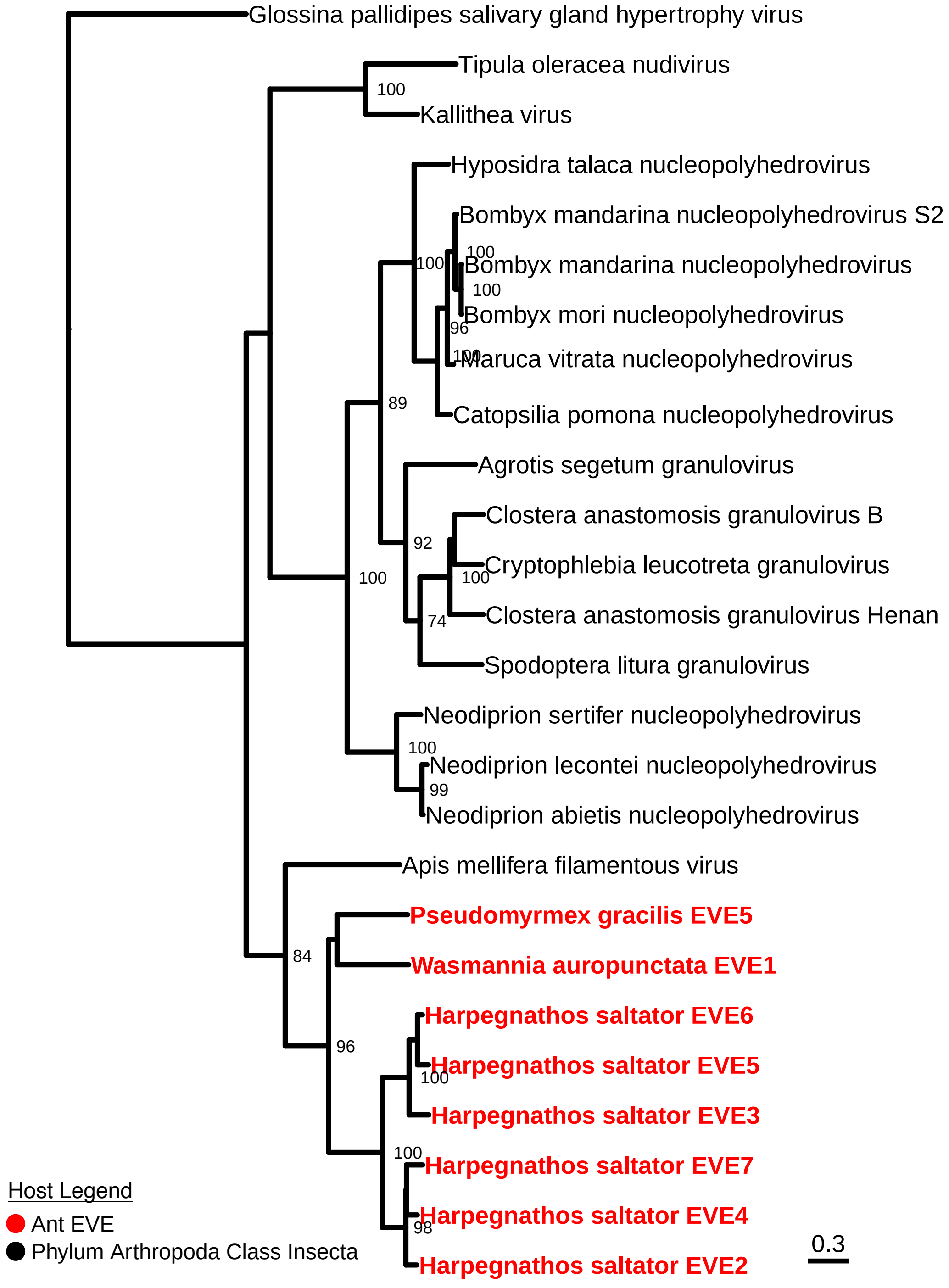

SF-19: Baculoviridae PIF-2 Phylogeny

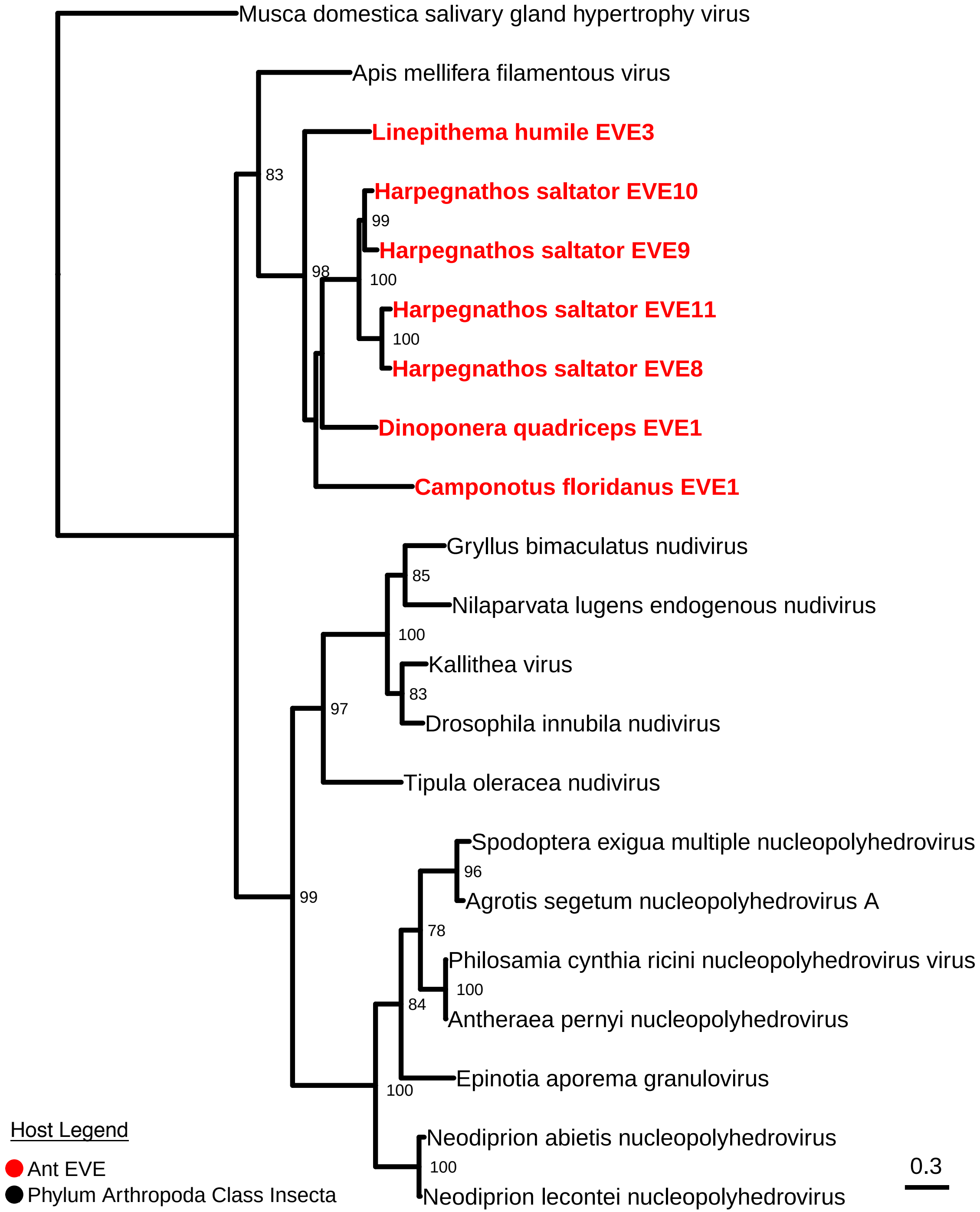

SF-20: Baculoviridae PIF-3 Phylogeny

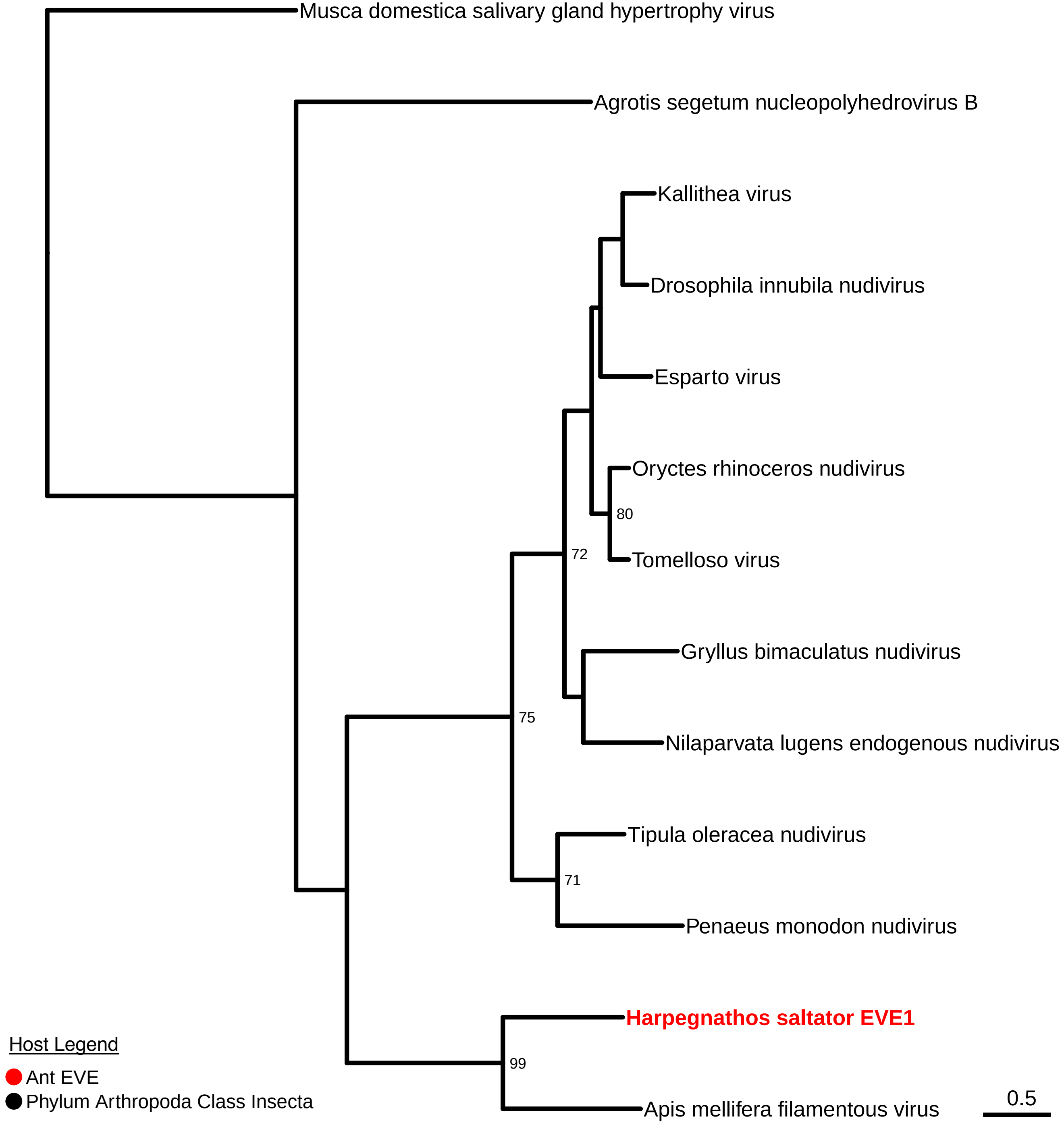

SF-21: Entomopoxvirus Tryptophan protein Phylogeny

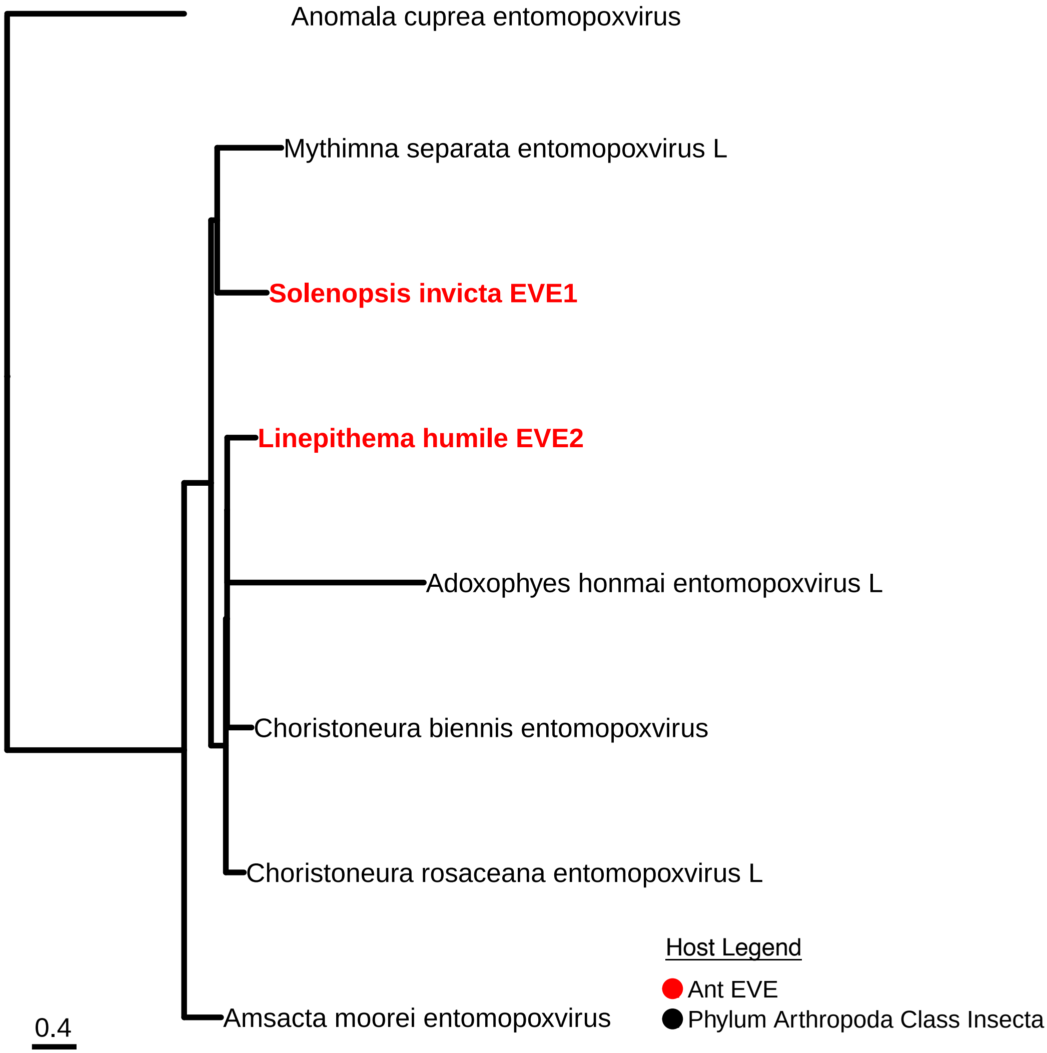

SF-22: Entomopoxvirus RNA polymerase Phylogeny

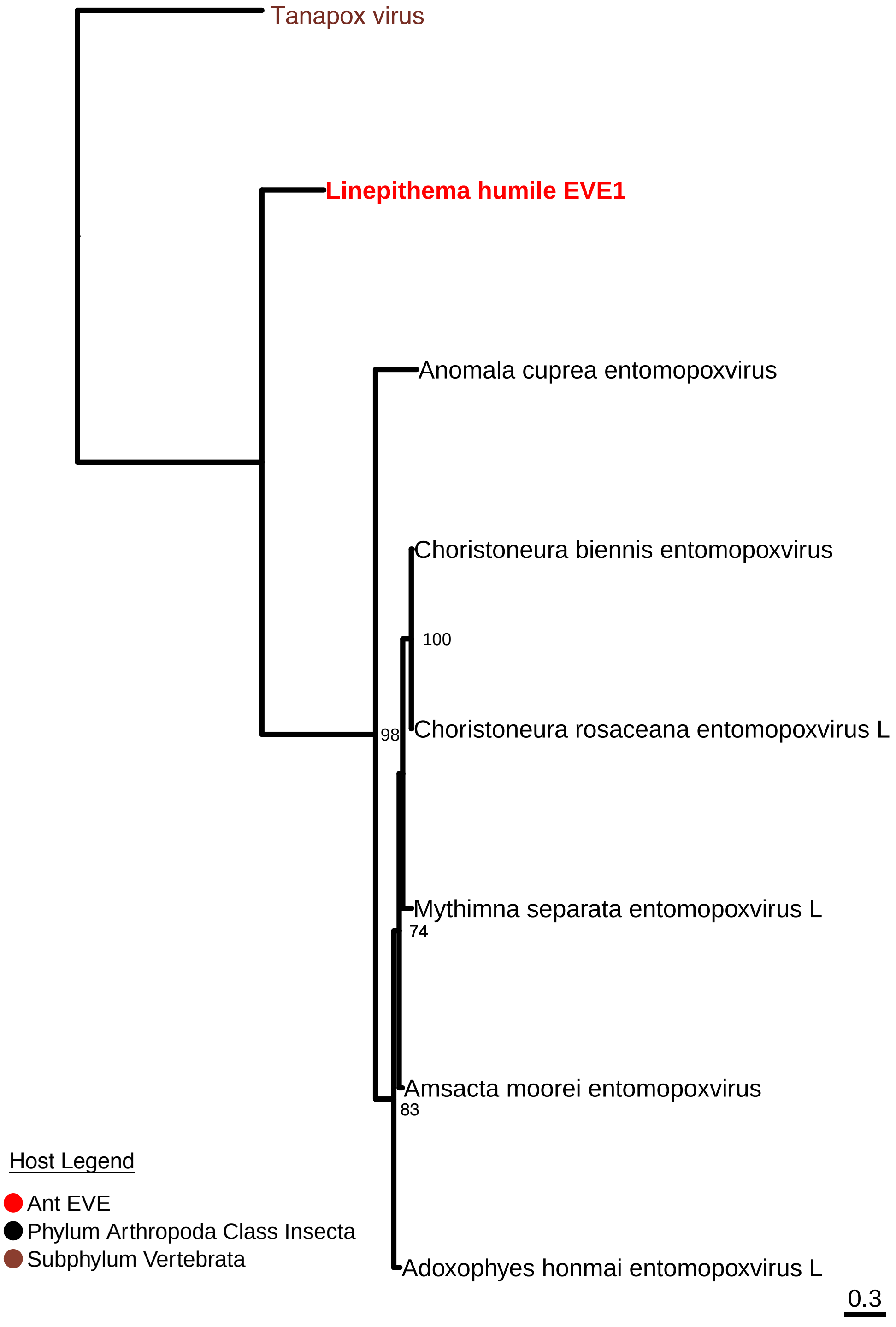

SF-23: Polydnaviridae PoxA32 protein Phylogeny

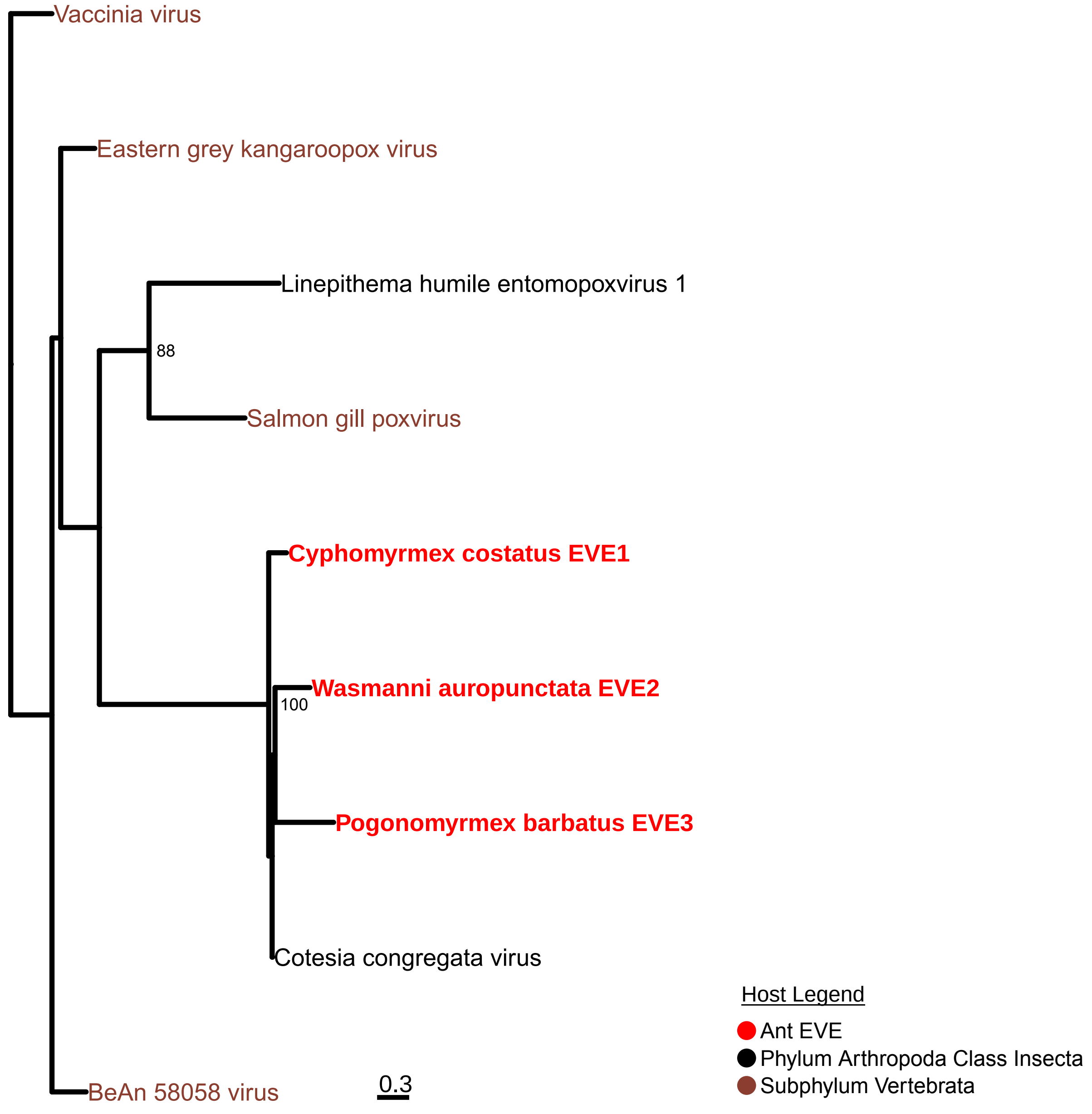

Supplement: Supplementary file 5 [file Data_Sheet_1.PDF]
